# A holistic framework for intradialytic hypotension prediction using generative adversarial networks-based data balancing

**DOI:** 10.1186/s12911-025-03094-5

**Published:** 2025-07-10

**Authors:** Hsuan-Ming Lin, JrJung Lyu

**Affiliations:** 1 Institute of Information Management, National Cheng Kung University, Tainan, Taiwan; 2 Internal Medicine, Nephrology Division, An Nan Hospital, China Medical University, Tainan, Taiwan; 3 Department of Industrial and Information Management, National Cheng Kung University, Tainan, Taiwan

**Keywords:** Generative adversarial networks, Intradialytic hypotension, Data balancing, Prediction model

## Abstract

**Background:**

Intradialytic Hypotension (IDH) is a frequent complication in hemodialysis, yet predictive modeling is challenged by class imbalance. Traditional oversampling methods often struggle with complex clinical data. This study evaluates an enhanced conditional Wasserstein Generative Adversarial Network with Gradient Penalty (CWGAN-GP) framework to improve IDH prediction by generating high-utility synthetic data for balancing.

**Methods:**

A CWGAN-GP was developed using multi-level hemodialysis data. Following rigorous preprocessing, including a strict temporal train-test split, the CWGAN-GP generated minority class samples exclusively on the training data. eXtreme Gradient Boosting (XGBoost) models were trained on the original imbalanced data and datasets balanced using the proposed CWGAN-GP method, benchmarked against traditional Synthetic Minority Over-sampling Technique(SMOTE) and Adaptive Synthetic Sampling Approach(ADASYN) balancing. Performance was evaluated using metrics sensitive to imbalance (e.g., Precision-Recall Area Under the Curve) and statistical comparisons, with SHapley Additive exPlanations (SHAP) analysis for interpretability.

**Results:**

The study population consisted of 40 chronic hemodialysis patients (45% male, mean age 66.30$\:\pm$ 10.68 years). An initial dataset, where intradialytic hypotension (IDH) events occurred in 14.85% of records (19,124 instances overall), was temporally split (75:25 ratio). This yielded an Original Training dataset of 95,856 samples (14.73% IDH rate) and a test set (15.21% IDH rate). From this Original Training dataset, a Generative Adversarial Network (GAN) was employed to construct a balanced dataset comprising 163,470 samples. The GAN Balanced dataset yielded the highest predictive performance, demonstrating statistically significant improvements over the Original Training dataset across metrics, including Precision-Recall Area Under the Curve (PR-AUC) (mean 0.735 vs 0.724) and Accuracy (mean 0.900 vs 0.892). In contrast, the GAN Augmented dataset (191,712 samples) showed mixed results (improved Accuracy/F1, decreased Receiver Operating Characteristic Curve Area Under Curve (ROC-AUC)/PR-AUC). In comparison, ADASYN (163,326 samples) and SMOTE (163,470 samples) balanced datasets significantly underperformed on PR-AUC. SHAP analysis identified Dialysis Date (as a proxy for temporal patterns like day-of-week) and hemodynamic indicators (e.g., Systolic Diastolic Difference, Previous Systolic Pressure) as key IDH predictors.

**Conclusion:**

The proposed CWGAN-GP framework effectively balances complex hemodialysis data, leading to significantly improved and interpretable IDH prediction models compared to standard approaches. This work supports leveraging advanced generative models like GAN to overcome data imbalance in clinical prediction tasks, which is pending further validation.

**Supplementary Information:**

The online version contains supplementary material available at 10.1186/s12911-025-03094-5.

## Background

End-stage renal disease (ESRD) represents a substantial global public health challenge, affecting an estimated 850 million individuals worldwide, with approximately 2 million requiring dialysis or transplantation [1]. The burden is escalating, driven partly by increasing diabetes-related ESRD and an aging population [2]. Hemodialysis (HD), the primary treatment, is frequently complicated by intradialytic hypotension (IDH), a condition clinically defined as a significant drop in blood pressureGAN Augmented dataset, occurring in 5% to 40% of sessions [3]. Several factors are known to increase the risk of IDH. For instance, advanced age, diabetes mellitus, and uremia can impair peripheral venous return, while underlying cardiovascular comorbidities such as coronary artery disease and congestive heart failure may compromise cardiac contractility and the ability to augment cardiac output [4]. The high prevalence of hypertension and diabetes among dialysis patients contributes to the elevated incidence of IDH. Furthermore, emerging evidence also suggests that temporal factors, including seasonal variations and day-of-the-week patterns possibly linked to interdialytic intervals, might influence IDH occurrence [5–8]. IDH poses serious risks, including tissue hypoperfusion, end-organ damage [3], and markedly increased in-hospital mortality (44% vs. 19% in non-IDH patients) [9]. It also diminishes quality of life through symptoms like nausea and fatigue [10]. The strong link between intradialytic blood pressure instability and adverse outcomes, including cardiovascular morbidity and mortality, is well-documented [11–16], highlighting the critical need for improved IDH prediction and prevention.

Machine Learning (ML) shows significant advances in various fields [17–19]. In healthcare, ML techniques are increasingly applied to improve diagnostic capabilities and predictive accuracy across diverse medical conditions and risk management tasks [20–30]. Specifically for IDH prediction, various ML algorithms (e.g., Decision Trees, Support Vector Machine(SVM), eXtreme Gradient Boosting(XGBoost), Light Gradient Boosting Machine(LightGBM), Recurrent Neural Network(RNN), Logistic Regression) have demonstrated promising results on large datasets, achieving accuracies or Receiver Operating Characteristic Curve Area Under Curve(ROC-AUC) ranging from approximately 0.74 to 0.94 [31–33]. Despite these advances, significant limitations persist. Many models are developed using single-center datasets, restricting their generalizability due to variations in treatment protocols and patient populations across different clinical settings. Furthermore, model effectiveness is often hindered by common challenges associated with medical data, including issues of data quality and availability. These limitations underscore the necessity for innovative methods to enhance the reliability and applicability of IDH prediction models.

The reliance on single-center data often stems from systemic challenges inherent to data collection and integration within the hemodialysis field. These challenges include a fragmented care system distributed across numerous smaller centers, which complicates large-scale multi-center studies [34]; high patient heterogeneity in terms of demographics, comorbidities, and treatment goals, hindering data pooling and impacting generalizability [35]; and limitations in the granularity of data available in larger registries, which may lack key real-time clinical variables necessary for specific research questions [36]. Collectively, these factors make assembling large, representative, high-quality datasets exceptionally difficult, often restricting research to single-center studies with consequent limitations in model generalizability [29].

Stemming from these broader systemic issues, specific data characteristics like data scarcity and class imbalance further hinder the development of robust ML models for IDH prediction. Regulatory constraints and variations in data standards across institutions can compound these problems. Critically, the relative rarity of IDH events compared to non-IDH sessions leads to highly imbalanced datasets. This imbalance introduces bias into model training, often reducing predictive performance for the minority class(IDH class). Traditional oversampling techniques, such as the Synthetic Minority Over-sampling Technique (SMOTE) and Adaptive Synthetic Sampling Approach(ADASYN), attempt to mitigate this by generating synthetic minority samples using interpolation-based strategies [37, 38]. However, these methods often struggle with the complexity inherent in medical data, potentially leading to issues like over-generalization, amplification of noise, or blurring of the decision boundary between classes [39–42].

Generative Adversarial Networks, introduced by Goodfellow et al. (2014) [43], offer a promising alternative to address these limitations. An GAN utilizes two competing neural networks: a generator (G) that learns to produce realistic synthetic data, and a discriminator (D) that learns to distinguish real data from the synthetic output. In contrast to methods relying on more straightforward interpolation, the adversarial learning process allows GAN to model complex, high-dimensional data distributions implicitly. This potentially enables them to capture nuanced inter-feature relationships and generate diverse synthetic samples that more faithfully reflect the true data distribution [43]. Furthermore, advanced variants like the Wasserstein Generative Adversarial Network with Gradient Penalty enhance training stability and performance, making GAN more applicable to challenging datasets, including those with significant class imbalance [44]. This capability to generate high-fidelity synthetic data makes GAN a valuable tool for addressing data scarcity and class imbalance, potentially enhancing machine learning model performance in various predictive tasks [33, 45].

Therefore, this study aims to address the limitations of existing IDH prediction models, particularly restricted generalizability and degraded performance due to class imbalance and data scarcity. By leveraging the potential of Generative Adversarial Networks to generate high-fidelity synthetic hemodialysis data, we seek to enhance the robustness and accuracy of subsequent prediction models. This research focuses on developing and evaluating a GAN-based framework to effectively augment imbalanced hemodialysis datasets, ultimately contributing to more reliable and clinically relevant AI-driven solutions for IDH prediction and improved patient management.

## Materials and methods

### Research framework

**Fig. 1:**
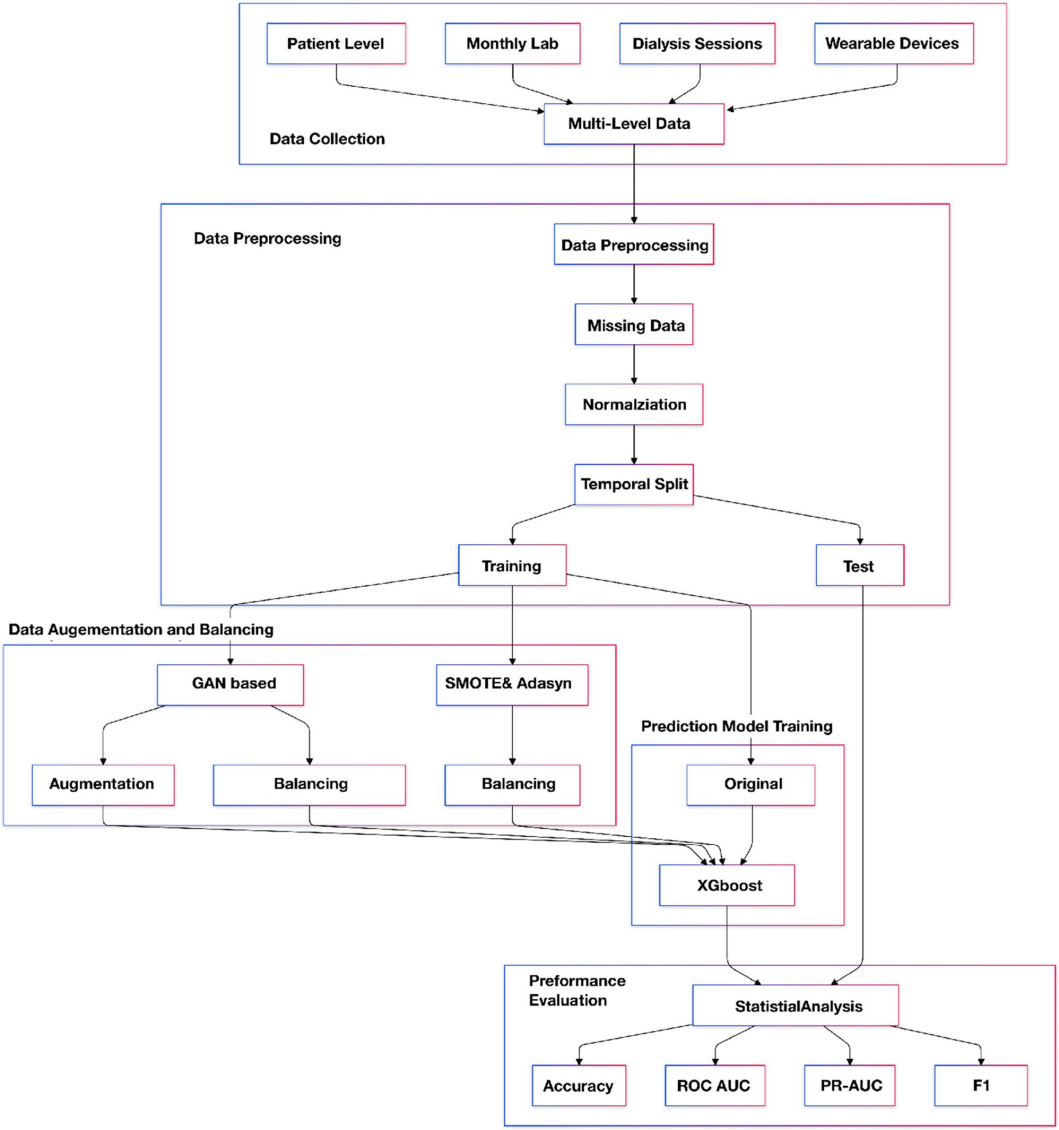
The workflow of the study

A holistic framework is proposed to validate the potential of GAN-based data-balancing methods over traditional statistical approaches in IDH prediction tasks (Fig. 1)

. The proposed framework consists of five main stages: Data Collection, Data Preprocessing, Data Generation, Prediction Model Training, and Effectiveness Evaluation and Statistical Analysis.

In the data collection stage, a multi-level data collection system encompassing four primary levels of clinical data was established. The first level comprises patient-level data, including demographic characteristics, primary diagnoses, chronic comorbidities, and other basic clinical information. The second level contains monthly examination data, recording periodic laboratory test results. The third level consists of per-dialysis session data, collecting real-time monitoring parameters during dialysis. The fourth level includes wearable device data, providing continuous physiological monitoring during dialysis sessions. Inclusion and exclusion criteria were established for participant selection, and the study protocol received Institutional Review Board (IRB) approval to ensure ethical compliance.

Systematic data cleaning and normalization procedures were employed in the data preprocessing stage. To ensure data quality and enhance model training effectiveness, records with missing data were removed, retaining only complete cases. Considering the potential non-normal distribution characteristics of features and sensitivity to outliers, the Robust Tanh Scaler was employed for feature normalization. This method centers data using the median and scales using the interquartile range (IQR), demonstrating less sensitivity to outliers compared to traditional Z-score standardization. A temporal split strategy was adopted to simulate real clinical scenarios, dividing the dataset into training and testing sets at a 75:25 ratio. This approach maintains temporal continuity, aiming to better reflect the model’s actual predictive capabilities in a prospective setting.

In the dataset generation stage, an improved conditional Wasserstein GAN architecture was applied to generate synthetic minority class data [44]. This architecture combines residual connections and spectral normalization techniques. Specifically, the generator adopts a deep residual network structure with a 256-dimensional input layer and two residual blocks, while the discriminator utilizes a three-layer feedforward neural network incorporating spectral normalization. During training, the Wasserstein distance served as the optimization objective, complemented by gradient penalty mechanisms to help enforce the Lipschitz continuity condition for the discriminator. For comparison with traditional balancing techniques, the ADASYN algorithm was selected to address class imbalance; ADASYN automatically determines the number of synthetic samples needed based on the local distribution density of minority class samples [46]. Multiple evaluation methods were employed to assess the quality of the generated synthetic data, including statistical metrics such as the Wasserstein distance(WD) and Kullback–Leibler divergence (KLD), comparing the distribution of generated samples to that of real samples within the minority class.

In the prediction model training stage, XGBoost was utilized as the primary prediction model. Multiple experiments were conducted using different hyperparameter settings to validate performance differences across various dataset balancing scenarios. Parameters varied included the maximum number of decision trees (ranging from 80 to 130 with an interval of 10) and the maximum tree depth (ranging from 8 to 16 with an interval of 2), resulting in 30 different parameter combinations tested for each dataset configuration. The datasets evaluated included: the Original Training data (imbalanced), and datasets balanced using SMOTE Balanced, ADASYN Balanced, GAN Augmented, and GAN Balanced approaches.

In the effectiveness evaluation and statistical analysis stage, multiple statistical methods were employed for analysis and comparison of model performance across the different dataset balancing approaches. 1-way Analysis of Variance(ANOVA) was used to evaluate overall differences in performance metrics between the methods, with the $\eta^2$ effect size calculated to assess practical significance. Pairwise comparison analyses were subsequently conducted using Cohen’s d effect size and the Bonferroni correction for multiple comparisons to identify specific differences between pairs of methods. Finally, visualization methods such as box plots were utilized to intuitively present the distribution characteristics of various performance metrics (including Accuracy, F1-score, ROC-AUC, and PR-AUC across the tested conditions.

### Data collection

This retrospective cohort study was conducted at the Nephrology Department of a regional medical center, utilizing data collected from September 2019 onwards. Patient data were retrospectively gathered from the hospital’s electronic health records (EHR) system following approval from the Institutional Review Board of Tainan Municipal An-Nan Hospital (IRB REC number: TMANH107–REC006CR2, approved Aug. 3, 2020). In view of the retrospective design of this study and the fact that all patient data used were anonymized and de-identified, the Institutional Review Board (IRB) approved the waiver of informed consent. All original data collected for this study were stored in the hospital’s data warehouse within the information system. A de-identification process was applied, which included removing patient names, dates of birth, medical record numbers, and other identifiable information before transforming the data for research use. Dates of birth were converted to patient ages, and medical record numbers were re-coded.

**Inclusion criteria** were defined as follows: (1) adult patients aged 18 to 90 years; (2) undergoing regular hemodialysis treatment for at least three months prior to enrollment; (3) receiving thrice-weekly dialysis sessions consistently; and (4) having complete and accessible clinical data records within the EHR system.

**Exclusion criteria** comprised: (1) cases with incomplete records for key clinical indicators necessary for IDH prediction modeling; (2) patients with irregular dialysis treatment frequency or significant schedule changes during the study period; and (3) patients who received a kidney transplant or converted to peritoneal dialysis during the data collection timeframe.

Data were collected across four primary levels reflecting different timescales and characteristics: patient-level baseline data (e.g., demographics, comorbidities), monthly examination results (e.g., laboratory tests), per-dialysis session parameters (e.g., real-time machine monitoring), and continuous wearable device signals obtained during dialysis. A detailed list of the variables included is provided in Supplementary Material 1.

This multi-level data collection strategy aimed to create a comprehensive dataset integrating stable baseline traits with dynamic physiological responses, facilitating detailed IDH risk modeling.

### Data preprocessing

The compiled multi-level dataset, containing diverse static and dynamic clinical features, underwent rigorous preprocessing to ensure data quality, consistency, and suitability for model training [47, 48]. Key steps included handling missing data, time-series feature engineering, temporal partitioning, and feature scaling. Specifically for handling missing data, to ensure data quality and improve model training effectiveness, we removed records containing missing key data and only retained complete cases for analysis. It is important to emphasize that no imputation of missing data using previous values, as sometimes employed in time-series analyses, was performed in this study. This approach of using only complete cases was also intentionally chosen in conjunction with our study’s use of time series splitting to mitigate potential data leakage issues that could arise from imputation across or within time-ordered records. No explicit feature selection method was applied prior to model training; the comprehensive feature set was used, relying on the XGBoost algorithm and subsequent SHAP analysis to determine feature relevance.

**Time-series feature engineering** The comprehensive feature set included the ‘Dialysis Date’, initially recorded in YYYY-MM-DD format. This date was first converted into a numerical feature representing the number of days elapsed since the earliest dialysis session in our dataset. This resulted in a monotonically increasing integer value. To incorporate temporal information for the prediction task (predicting the subsequent measurement’s IDH risk), key time-series features like blood pressure were processed using feature engineering and specifically, lagged variables representing values from the previous one or two measurement time points (e.g., ‘Previous Systolic Pressure’, ‘Second Previous Systolic Pressure’ were created. Crucially, these lagged features were calculated strictly using data from within the same dialysis session. Records where necessary lagged features could not be generated (e.g., at the very beginning of a session) might be excluded at this stage or handled appropriately during modeling. These lagged features were included alongside other contemporaneous measurements in the input for the subsequent modeling steps.

**Temporal data partitioning (train-test split)** Following initial cleaning and time-series feature engineering, the dataset was divided chronologically based on session dates to prevent data leakage and simulate a realistic clinical prediction scenario. The training set comprised the first 75% of the sessions, and the testing set contained the most recent 25%. Because the split separates entire dialysis sessions, the calculation of within-session lagged features for the training set cannot incorporate any information from test set sessions, thereby preventing data leakage through this feature engineering step. This temporal split approach is essential for time-series medical data analysis. It ensures the model learns only from past information to predict future outcomes and preserves inherent temporal patterns.

**Feature standardization** After the temporal split, features were standardized using the Robust Tanh Scaler [49]. This 2-step method first robustly scales features using the median and interquartile range, making it less sensitive to outliers, and then applies a hyperbolic tangent (tanh) transformation to map values into the bounded range of (−1, 1). This approach provides resilience to outliers while producing bounded outputs suitable for subsequent GAN modeling [49–51]. Crucially, the scaler was fitted only on the training data and then used to transform both the training and test sets.

### Augmentation and balancing data generation

#### GAN-based data augmentation and balancing

A conditional Wasserstein GAN with Gradient Penalty (cWGAN-GP) architecture [44, 52] was employed to generate synthetic hemodialysis session data exclusively on the preprocessed (split and scaled) training dataset, explicitly targeting the minority class (IDH). The cWGAN-GP framework was chosen for its advantages in training stability and its ability to generate high-fidelity, diverse samples, particularly for complex and imbalanced medical datasets, compared to standard GAN architectures. Key features include the use of the Wasserstein distance, providing a more stable loss signal, and a gradient penalty mechanism to enforce the Lipschitz constraint on the discriminator, mitigating common GAN training issues like mode collapse and vanishing gradients [44]. The conditional nature of the model allows targeted data generation based on class labels (IDH or non-IDH), facilitating precise dataset balancing.

The cWGAN-GP architecture comprises a generator (G) and a discriminator (D, also referred to as a critic). The generator G takes a random noise vector (z) drawn from a standard normal distribution and a condition vector (c, representing the desired class label) as input to produce synthetic data samples ($\tilde{x}$). The discriminator D takes either a real data sample (x) or a synthetic sample ($\tilde{x}$), along with the condition c, and outputs a Scaler value indicating the perceived realness of the input.

The cWGAN-GP training process involves iteratively updating the generator and discriminator networks. The discriminator is trained to maximize the difference between its output on real versus synthetic samples, while incorporating the gradient penalty term. The generator is trained concurrently to minimize this difference, effectively learning to produce samples that the discriminator deems realistic. The detailed structure and steps of the training algorithm are outlined in Table 1.

**Table 1:** cWGAN-GP training procedure

Step	Description
**Initialize:**	Generator$G$ and discriminator$D$ with random weights$\theta_G$ and$\theta_D$ using Kaiming initialization for linear layers.
**While**$\,\,\,G$ has not converged:	
	**For**$\,t = 1$ to 5 (n_critic steps):
	Load a batch of real data$\mathbf{x}$ and labels$\mathbf{y}$ from the training dataset ($P_r, P_y$).
	Generate random noise vectors$\mathbf{z} \sim \mathcal{N}(0,1)$ (dimension 128).
	Produce synthetic data using the generator:$\tilde{\mathbf{x}} = G(\mathbf{z}, \mathbf{y})$.
	Sample$\epsilon \sim U[0, 1]$.
	Compute interpolated samples:$\hat{\mathbf{x}} = \epsilon \cdot \mathbf{x} + (1 - \epsilon) \cdot \tilde{\mathbf{x}}$.
	Calculate the discriminator loss (incorporating gradient penalty):
	$$\begin{array}{l}L_D = - \text{mean}(D(\mathbf{x}, \mathbf{y})) + \text{mean}(D(\tilde{\mathbf{x}}, \mathbf{y})) + \\\lambda \cdot \text{mean}\left( \left( \left\| \nabla_{\hat{\mathbf{x}}} D(\hat{\mathbf{x}}, \mathbf{y}) \right\|_2 - 1 \right)^2 \right)\end{array}$$
	Update discriminator weights$\theta_D$ by descending the gradient$\nabla_{\theta_D} L_D$ using RMSprop optimizer.
	**End For**
	Generate new random noise vectors$\mathbf{z} \sim \mathcal{N}(0,1)$.
	Generate new synthetic data$\tilde{\mathbf{x}} = G(\mathbf{z}, \mathbf{y})$.
	Calculate generator loss:$L_G = - \text{mean}(D(\tilde{\mathbf{x}}, \mathbf{y}))$.
	Update generator weights$\theta_G$ by descending the gradient$\nabla_{\theta_G} L_G$ using RMSprop optimizer.
	Adjust learning rates for$G$ and$D$ using a step scheduler.
**End While**	
**Return** Trained Generator $G$	

*Note P*_*r*_, *P*_*y*_: distributions from training loader. z: 128-dim $\mathcal{N}$ (0, 1). λ: gradient penalty weight = 5. Optimizer: RMSprop. Learning rates: α_*G*_ = 0.001, α_*D*_ = 0.002. Scheduler: StepLR (step size 30, gamma 0.9). Batch size *m* determined by loader (e.g., 1024 used in training visualization). Training on NVIDIA GeForce RTX 4060 Ti GPU

#### SMOTE and ADASYN data balancing

To compare the performance of the proposed GAN-based data balancing with traditional oversampling techniques, two widely used methods were applied as benchmarks: SMOTE [53] and ADASYN (Adaptive Synthetic Sampling Approach) [38]. These methods were applied exclusively to the preprocessed (split and scaled) training dataset to generate two distinct balanced datasets for comparative evaluation.

SMOTE [53], a widely recognized oversampling technique applied across various domains, including medical data preprocessing [47, 54], operates by generating synthetic minority samples through interpolation. It identifies k-nearest neighbors within the minority class for each minority instance. It creates new synthetic samples along the line segments connecting the instance to some of its chosen neighbors in the feature space. This process is repeated until the desired class balance is achieved.

ADASYN [38] builds upon SMOTE by introducing an adaptive approach. It generates more synthetic data for minority class instances that are considered “harder to learn,” typically those located closer to the decision boundary with the majority class. This adaptivity is achieved by assigning weights to minority instances based on the prevalence of majority class samples in their neighborhood. Instances with higher weights (indicating greater learning difficulty) receive proportionally more synthetic samples generated using a SMOTE-like interpolation mechanism, thus focusing the balancing effort on more challenging regions of the feature space.

Using both SMOTE and ADASYN allows for a comprehensive evaluation, comparing the effectiveness of these standard and adaptive interpolation-based oversampling strategies against the proposed GAN-based approach.

#### Data distribution similarity assessment

To evaluate the fidelity of generated synthetic data in replicating the distribution of the original real data, three complementary statistical distance metrics were employed: Wasserstein Distance, Kullback-Leibler Divergence, and Jensen-Shannon Divergence(JSD). These metrics provide distinct perspectives on distributional similarity and are widely utilized in assessing generative models [43, 55, 56].

1. Wasserstein Distance: Also known as the Earth Mover’s Distance, WD measures the minimum cost required to transform one probability distribution into another. It is particularly advantageous when distributions have limited overlap, a situation where other divergence measures might be uninformative [52]. A lower WD indicates greater similarity between the distributions being compared.
2. Kullback-Leibler Divergence: KLD quantifies the information loss when one probability distribution (Q) is used to approximate another (P), effectively measuring their relative entropy [57]. KLD is asymmetric (i.e., KL(P||Q)$\neq$ KL(Q||P)) and can become infinite if the supports of the two distributions do not sufficiently overlap [58]. A lower KLD value signifies greater similarity.
3. Jensen-Shannon Divergence: JSD is a symmetrized and bounded adaptation of KLD, providing a stable measure of similarity between two probability distributions [59, 60]. Its calculation is based on the KLD between each distribution and their average mixture distribution. JSD is symmetric, non-negative, and always yields a finite value. A lower JSD indicates greater distributional similarity.

To statistically compare these quantitative distance metrics (WD, KLD, JSD) across the four different data generation/balancing methods (GAN Augmented, Adasyn Balanced, SMOTE Balanced, and GAN Balanced dataset), the non-parametric Kruskal-Wallis test [61] was employed. This rank-based test was selected because it does not require assumptions of normality or homoscedasticity. If the Kruskal-Wallis test indicated a significant overall difference among the methods (${p}< 0.05$), post-hoc pairwise comparisons were subsequently performed using Mann-Whitney U tests [62] to compare the methods directly against each other. The Bonferroni correction was applied to adjust for the six pairwise comparisons among the four methods, setting the significance level at$\alpha$ = 0.05 / 6$\approx$ 0.0083 [63].

### Prediction model development and performance evaluation

For the predictive modeling of intradialytic hypotension, XGBoost was selected as the primary prediction model. XGBoost is a gradient boosting algorithm widely recognized for its high predictive performance, efficiency, and robustness, particularly in handling complex, high-dimensional, and potentially imbalanced datasets common in medical applications [64]. Its selection was further motivated by its established use in medical prediction tasks and inherent capabilities useful for clinical data, such as effectively handling missing values [22, 32].

To evaluate the impact of different data balancing methods (Original Training, GAN Augmented, ADASYN Balanced, SMOTE Balanced, GAN Balanced) on the predictive performance of XGBoost and ensure the robustness of findings across various model configurations, a systematic parameter evaluation was conducted. Instead of optimizing hyperparameters solely for one dataset version, two key hyperparameters influencing model complexity were varied: n_estimators (number of trees) and max_depth (maximum tree depth), based on prior research and experimentation [65]. A grid search-like approach explored combinations within predefined ranges: n_estimators from 80 to 130 (step 10: 80, 90,…, 130) and max_depth from 8 to 16 (step 2: 8, 10,…, 16). These 30 distinct parameter combinations were applied when training XGBoost on each dataset version. The primary purpose of evaluating these combinations was to verify if observed relative performance differences between the dataset balancing methods were consistent and statistically significant across a range of representative model complexities, rather than being an artifact of a single parameter setting. The Area Under the Receiver Operating Characteristic curve (ROC AUC) served as the primary evaluation metric for comparing model performance across these conditions, chosen for its robustness, especially in imbalanced datasets. Other XGBoost parameters were held constant at standard values (detailed in Table 2) to ensure a consistent basis for comparison.

**Table 2:** Xgboost hyperparameter configuration

Parameter	Value(s)	Description / Rationale
objective	binary:logistic	Objective for binary classification (outputting probabilities)
eval_metric	[‘logloss’, ‘auc’]	Metrics for evaluating model performance
booster	gbtree	Tree-based booster (Standard)
learning_rate	0.3	Step size shrinkage (Standard Default)
n_estimators	80, 90, 100, 110, 120, 130	(Varied) Number of boosting rounds/trees evaluated for robustness testing
max_depth	8, 10, 12, 14, 16	(Varied) Maximum depth of a tree evaluated for robustness testing
min_child_weight	1	Minimum sum of instance weight needed in a child (Default)
gamma	0	Minimum loss reduction required for split (Default)
subsample	1	Subsample ratio of the training instance (Default: No subsampling)
colsample_bytree	1	Subsample ratio of columns when constructing each tree (Default)
colsample_bylevel	1	Subsample ratio of columns for each level (Default)
colsample_bynode	1	Subsample ratio of columns for each node (split) (Default)
reg_alpha	0	L1 regularization term on weights (Default: No L1 reg.)
reg_lambda	1	L2 regularization term on weights (Default: L2 reg. = 1)
scale_pos_weight	1	Controls balance of positive/negative weights (Default: No re-weighting, appropriate for comparing data balancing methods)
tree_method	hist	Tree construction algorithm (Efficient histogram-based)
max_bin	256	Maximum number of discrete bins for features (hist method) (Default)
grow_policy	depthwise	Controls how nodes are added (Default: Depthwise)
random_state	42	Random number seed for reproducibility

Finally, model performance was evaluated on the held-out test set using four complementary metrics. Accuracy (Eq.1) measured overall correctness using true positives (TP), true negatives (TN), false positives (FP), and false negatives (FN). Given the potential class imbalance, metrics focusing on the minority class were prioritized: the F1-score (Eq.4), which is the harmonic mean of precision (Eq.2) and recall (Eq.3); the Receiver Operating Characteristic Area Under the Curve (ROC AUC), assessing classification discrimination ability; and the Precision-Recall Area Under the Curve (PR-AUC), which is particularly informative for evaluating performance on imbalanced datasets.

1 $$\text{Accuracy} = \frac{TP + TN}{TP + TN + FP + FN}$$

2 $$\text{Precision} = \frac{TP}{TP + FP}$$

3 $$\begin{aligned}\text{Recall} = \frac{TP}{TP + FN} \quad (&\text{also\:known\:as\:Sensitivity} \cr& \text{or\:True\:Positive\:Rate})\end{aligned}$$

4 $$\text{F1-score} = 2 \times \frac{\text{Precision} \times \text{Recall}}{\text{Precision} + \text{Recall}}$$

### Statistical analysis framework

A multi-level statistical framework was implemented to rigorously evaluate the impact of different data balancing strategies on IDH prediction model performance, comparing models trained on five distinct datasets: the Original Training data (imbalanced), and datasets balanced using SMOTE Balanced, ADASYN Balanced, GAN Augmented, and GAN Balanced approaches. This framework aimed to assess both the statistical significance and practical importance of performance differences. Key stages included overall comparison using ANOVA, specific pairwise comparisons, and visual analysis using box plots.

One-way ANOVA was employed to detect overall statistically significant differences in each performance metric (Accuracy, F1-score, ROC AUC, PR-AUC) across the models trained on the five datasets [3, 18, 20]. A significance level of$\alpha = 0.05$ was used. To quantify the practical significance of any overall differences, the omega-squared ($\omega^2$) effect size was calculated, representing the proportion of variance in the performance metric explained by the dataset type (interpreted as small$\geq$ 0.01, medium$\geq$ 0.06, large$\geq$ 0.14).

If ANOVA indicated a significant overall difference (${p}< 0.05$), post-hoc pairwise comparisons using Mann-Whitney U tests were performed to identify specific differences between pairs of the five datasets. The Bonferroni correction was applied to adjust for the 10 pairwise comparisons, setting the significance level for pairwise tests at$\alpha = 0.05 / 10 = \mathbf{0.005}$ [63]. To assess the practical significance of pairwise differences, Cohen’s d effect size was calculated for each comparison, interpreted using standard guidelines (small$|d| \geq$ 0.2, medium$\geq$ 0.5, large$\geq$ 0.8) [66, 67].

Finally, box plots were generated to visually represent the distribution characteristics of each performance metric across the different data balancing methods. Box plots effectively display the median, interquartile range, and potential outliers for each group, aiding in intuitively understanding the performance differences and visually assessing the variability and distribution characteristics of the results.

Through this multi-level statistical analysis framework, which combines ANOVA, posthoc pairwise comparisons with effect sizes and Bonferroni correction, and box plot visualizations, we aimed to provide a robust, comprehensive, and interpretable evaluation of the impact of different data balancing strategies on IDH prediction model performance, addressing both statistical significance and practical relevance.

### SHAP analysis

To enhance the interpretability of our IDH prediction models, particularly those trained with GAN-balanced datasets, we employed SHapley Additive exPlanations analysis [68]. SHAP, a game-theoretic approach to explainable AI, provides a robust framework for quantifying each feature’s contribution to individual predictions, thereby increasing model transparency and addressing the ‘black box’ nature of complex models like XGBoost. This analysis is crucial for understanding model decision-making, potentially validating whether essential feature relationships are preserved in GAN-generated data, and aiming to derive clinically relevant insights beyond predictive accuracy alone.

SHAP values were calculated using the TreeExplainer algorithm, which is specifically optimized for tree-based models such as XGBoost. Feature importance and effects were visualized using summary plots. These plots rank features based on their mean absolute SHAP values across all samples and illustrate the directionality of each feature’s impact (positive or negative) on the IDH risk prediction. This SHAP analysis aimed to provide interpretable and clinically meaningful insights into the factors driving IDH prediction in our models.

## Results

### Data collection and data characteristics

The initial dataset for this study was compiled from a single medical center’s hemodialysis database, encompassing data from 46 participants across 1,325 dialysis sessions. This included diverse data types such as 11,315 blood pressure measurements, 238,477 dialysis machine readings, 328 laboratory results, 1,361 nursing records, and 5,484,217 physiological data points from wearable devices collected across 593 participant instances.

A data cleaning stage was implemented to ensure data quality and feature completeness. Records associated with blood pressure measurements were excluded if key lagged features (e.g., previous blood pressure, next measured systolic blood pressure) were missing or if extreme outlier values were present (4,203 exclusions). Additionally, records lacking essential blood pressure data were removed (109,736 exclusions). Furthermore, participants lacking corresponding dialysis machine data alongside their blood pressure readings were excluded, reducing the final cohort to 40 participants. The resulting analysis dataset comprised 128,741 curated records (representing distinct measurement time points across sessions), linked to these 40 participants and incorporating 7,112 specific blood pressure measurement events.

Within this final dataset, Intradialytic Hypotension (IDH) events occurred in 14.85% of the records (19,124 instances). This dataset was then split temporally into training and testing sets based on session dates at a 75:25 ratio, with IDH event proportions of 14.73% in the training set and 15.21% in the testing set. The final study population of 40 chronic hemodialysis patients consisted of 45% male, with a mean age of 66.30$\pm$ 10.68 years; Other measurement-based characteristics and statistical test results are reported in Table 3.

**Table 3:** Baseline characteristics and univariate GEE P-values, stratified by outcome

Variables	No Event (Outcome = 0)	Event (Outcome = 1)	*P*-value
	(N = 109617)	(N = 19124)	(Univar. GEE)
**Patient Baseline Data**			
Male	55648 (50.8%)	8741 (45.7%)	0.519
Diabetes Mellitus Status	68188 (62.2%)	9205 (48.1%)	0.048
Hypertension Status	4219 (3.8%)	189 (1.0%)	0.001
Cardiothoracic Ratio Binary	38246 (34.9%)	4965 (26.0%)	0.185
Midodrine Administration	46233 (42.2%)	7911 (41.4%)	0.918
Patient Age	66.6 $\pm$ 10.2	68.5 $\pm$ 11.3	0.276
**Nursing Records Data**			
Venous Needle Gauge	16.0 $\pm$ 0.9	15.9 $\pm$ 1.4	0.024
Arterial Needle Gauge	16.0 $\pm$ 0.8	16.0 $\pm$ 1.2	0.744
Fall Risk Score	5.2 $\pm$ 1.8	5.5 $\pm$ 1.8	0.257
Erythropoietin Dosage	1660.2 $\pm$ 1300.5	1482.5 $\pm$ 1257.2	0.092
Dialysate Calcium Concentration	2.6 $\pm$ 0.3	2.7 $\pm$ 0.3	0.509
Oral Fluid Intake	32.7 $\pm$ 105.4	39.5 $\pm$ 116.5	0.262
Dry Weight	64.0 $\pm$ 19.1	65.2 $\pm$ 15.8	0.564
Pre Dialysis Weight Net	69.0 $\pm$ 15.1	68.6 $\pm$ 15.0	0.857
Ultrafiltration Machine Setting	2.3 $\pm$ 0.8	2.5 $\pm$ 0.9	0.186
Post Dialysis Weight Net	66.3 $\pm$ 16.0	65.7 $\pm$ 15.9	0.780
Actual Ultrafiltration	2.5 $\pm$ 3.3	2.5 $\pm$ 3.2	0.577
Arterial Puncture Count	1.0 $\pm$ 0.1	1.0 $\pm$ 0.1	0.577
Venous Puncture Count	1.0 $\pm$ 0.1	1.0 $\pm$ 0.1	0.692
**Hemodialysis Machine Data**			
Venous Pressure	145.3 $\pm$ 36.8	140.7 $\pm$ 37.6	0.342
Arterial Pressure	0.5 $\pm$ 1.4	0.5 $\pm$ 1.4	0.982
Temperature	64.3 $\pm$ 16.2	64.9 $\pm$ 17.1	0.734
Actual Blood Flow	250.3 $\pm$ 38.7	242.2 $\pm$ 42.9	0.099
Treatment Temperature	36.0 $\pm$ 0.4	35.9 $\pm$ 0.5	0.097
Actual Temperature	36.0 $\pm$ 0.8	35.9 $\pm$ 0.6	0.143
Dialysate Conductivity	14.0 $\pm$ 0.3	14.0 $\pm$ 0.2	0.598
Ultrafiltration Rate	0.6 $\pm$ 0.2	0.7 $\pm$ 0.2	0.055
Ultrafiltration Target	2.4 $\pm$ 0.8	2.6 $\pm$ 0.9	0.061
Ultrafiltration Volume	1.1 $\pm$ 0.7	1.2 $\pm$ 0.8	0.026
Ultrafiltration Time	130.1 $\pm$ 57.2	125.0 $\pm$ 55.2	0.210
Dialysate Flow Rate	499.1 $\pm$ 47.1	497.8 $\pm$ 43.0	0.648
Target Sodium Concentration	1394.1 $\pm$ 12.5	1393.2 $\pm$ 12.8	0.497
Bicarbonate Concentration	−0.0 $\pm$ 0.6	0.2 $\pm$ 0.6	0.043
Effective Blood Flow	249.9 $\pm$ 38.7	241.9 $\pm$ 42.3	0.111
Accumulated Blood Volume	1583.4 $\pm$ 1631.8	1714.1 $\pm$ 1615.0	0.490
Ultrafiltration Profile	80833 (73.7%)	15103 (79.0%)	0.223
Sodium Profile	91927 (83.9%)	16919 (88.5%)	0.210
Heparin Delivery Rate	11.9 $\pm$ 27.9	11.5 $\pm$ 42.3	0.870
**Laboratory Data**			
Alkaline Phosphatase	96.6 $\pm$ 56.9	104.7 $\pm$ 57.2	0.313
Alanine Aminotransferase	19.0 $\pm$ 13.8	22.3 $\pm$ 17.4	0.242
Albumin Bcg	4.2 $\pm$ 0.3	4.1 $\pm$ 0.3	0.028
Blood Urea Nitrogen	64.7 $\pm$ 18.6	66.2 $\pm$ 20.0	0.490
Carbon Dioxide	22.1 $\pm$ 2.1	22.1 $\pm$ 2.2	0.939
Creatinine	9.5 $\pm$ 2.0	9.4 $\pm$ 2.0	0.846
Mean Corpuscular Volume	90.6 $\pm$ 9.9	91.5 $\pm$ 8.9	0.407
Glomerular Filtration Rate	5.1 $\pm$ 1.2	5.0 $\pm$ 1.4	0.779
Glucose AC	157.9 $\pm$ 71.3	178.5 $\pm$ 75.1	0.046
Hemoglobin	10.6 $\pm$ 1.1	10.7 $\pm$ 1.2	0.340
Hematocrit	31.7 $\pm$ 3.5	32.1 $\pm$ 3.9	0.385
Potassium	4.6 $\pm$ 0.7	4.6 $\pm$ 0.7	0.786
Calcium	9.7 $\pm$ 0.6	9.6 $\pm$ 0.6	0.355
Sodium	136.2 $\pm$ 2.6	135.3 $\pm$ 3.0	0.047
Phosphorus	4.9 $\pm$ 1.3	5.0 $\pm$ 1.1	0.494
Platelet Count	200.8 $\pm$ 60.8	205.0 $\pm$ 57.2	0.428
Red Blood Cell Count	3.5 $\pm$ 0.6	3.5 $\pm$ 0.5	0.952
White Blood Cell Count	6.5 $\pm$ 2.3	6.8 $\pm$ 2.2	0.207
Mean Corpuscular Hb	30.3 $\pm$ 3.5	30.6 $\pm$ 3.1	0.443
Mean Corpuscular Hb Concentration	33.4 $\pm$ 1.5	33.4 $\pm$ 1.5	0.866
Red Cell Distribution Width	15.3 $\pm$ 1.8	15.3 $\pm$ 1.5	0.916
Globulin	2.6 $\pm$ 0.4	2.7 $\pm$ 0.4	0.121
**Vital Signs Data**			
Initial Systolic Pressure	155.9 $\pm$ 26.4	159.9 $\pm$ 35.4	0.512
Initial Diastolic Pressure	68.0 $\pm$ 13.3	67.0 $\pm$ 15.3	0.653
Systolic Diastolic Difference	73.1 $\pm$ 18.2	55.4 $\pm$ 21.5	0.001
Previous Systolic Pressure	136.7 $\pm$ 24.1	138.5 $\pm$ 36.9	0.753
Previous Mean Arterial Pressure	91.8 $\pm$ 16.6	89.8 $\pm$ 24.9	0.567
Previous Diastolic Pressure	65.3 $\pm$ 12.8	63.1 $\pm$ 19.8	0.360
Previous Pulse Rate	77.2 $\pm$ 12.9	80.2 $\pm$ 16.6	0.076
Previous Systolic Diastolic Difference	71.4 $\pm$ 18.8	75.4 $\pm$ 26.7	0.247
Second Previous Systolic	140.4 $\pm$ 24.5	139.6 $\pm$ 33.9	0.870
Ocare Heart Rate Mean	55.5 $\pm$ 36.0	58.0 $\pm$ 38.7	0.537
Ocare Heart Rate Cv	0.02 $\pm$ 0.02	0.02 $\pm$ 0.02	0.184
Ocare Oxygen Saturation Mean	65.1 $\pm$ 40.1	64.7 $\pm$ 40.8	0.919
Ocare Oxygen Saturation Cv	0.01 $\pm$ 0.01	0.01 $\pm$ 0.01	0.777

*Note* Data are presented as n (%) for binary variables and Mean ± Standard Deviation (SD) for continuous variables, stratified by outcome group (No Event: Outcome = 0, Event: Outcome = 1). N represents the number of hemodialysis sessions in each group. P-value is derived from univariate Generalized Estimating Equations (GEE) analysis for each variable against the intradialytic hypotension events, accounting for patient clustering (Independence covariance structure). See Supplyment Table 1 for detailed variable descriptions and units

Univariate Generalized Estimating Equations (GEE) analyses, accounting for patient clustering, were performed for each variable against the IDH outcome. Several variables showed a statistically significant association (${P}< 0.05$) with IDH events in these initial analyses. Detailed P-values derived from the GEE analysis are presented in Table 3.

### GAN training process, data generation and balance

As mentioned, a conditional Wasserstein GAN (cWGAN) architecture was adopted, which demonstrates robust convergence characteristics and stable training dynamics. The training process depicts the loss curves for the generator and discriminator networks over approximately 1900 iterations. The the training process and loss curves were showen in Supplementory Material 1.

### Dataset characteristics

The datasets used in this study were derived from an initial temporal split of the collected data. This split yielded an Original Training Set containing 95,856 samples (14.73% positive IDH rate) and a Test Set with 32,885 samples (15.21% positive IDH rate). Both exhibited inherent class imbalance. The Original Training Set was then used to create balanced datasets using different methods for performance comparison. Balancing with ADASYN resulted in the ADASYN Balanced Training Set (163,326 samples, 49.9% positive rate). The SMOTE Balanced Training Set contained 163,470 samples. Using our cWGAN-GP method, the GAN Balanced Training Set was created (163,470 samples). Statistics for these datasets, along with an intermediate dataset referred to as GAN Augmented Dataset (191,712 samples), are reported in Table 4.

**Table 4:** Summary statistics of datasets used in the study

Dataset	Total Samples	Positive Samples (IDH)	Positive Rate (%)
Original Training Dataset	95,856	14,121	14.73
Original Test Dataset	32,885	5,003	15.21
ADASYN Balanced Dataset	163,326	81,591	49.96
SMOTE Balanced Dataset	163,470	81,735	50.00
GAN Augmented Dataset	191,712	62,367	32.53
GAN Balanced Dataset	163,470	81,735	50.00

### Generated data quality assessment

Quantitative metrics were employed to evaluate the fidelity of the generated synthetic data in replicating the distribution of the original real data quantitatively. Three complementary statistical distance metrics were applied: Wasserstein Distance (WD), Kullback-Leibler Divergence (KLD), and Jensen-Shannon (JS) Divergence. These metrics provide distinct perspectives on distributional similarity [43, 55, 56].

Kruskal-Wallis tests followed by post-hoc Mann-Whitney U tests with Bonferroni correction were conducted to assess the statistical significance of the differences in these distance metrics across the data generation methods. Table 5 presents the Kruskal-Wallis test results, and Table 6 shows the pairwise comparison results, comparing the GAN Augmented, Adasyn Balanced, SMOTE Balanced, and GAN Balanced datasets.

**Table 5:** Kruskal-wallis test results for distribution similarity metrics

Metric	Kruskal-Wallis H	p-value
Wasserstein Distance	20.827	< 0.001 *
KL Divergence	162.830	< 0.001 *
JS Divergence	165.802	< 0.001 *

*Note* The Kruskal-Wallis H test was used to determine if there were statistically significant differences in the distribution similarity metrics (Wasserstein Distance, KL Divergence, and JS Divergence) between the different data generation methods (GAN Augmented, Adasyn Balanced, SMOTE Balanced, and GAN Balanced). A *p*-value less than 0.05 indicates a statistically significant difference

**Table 6:** Mann-Whitney U pairwise comparison results for distribution similarity metrics

Comparison	Wasserstein Distance	KL Divergence	JS Divergence
GAN Augmented vs. ADASYN Balanced	$$< $$ 0.001 *	$$< $$ 0.001 *	$$< $$ 0.001 *
GAN Augmented vs. SMOTE Balanced	$$< $$ 0.001 *	$$< $$ 0.001 *	$$< $$ 0.001 *
GAN Augmented vs. GAN Balanced	0.050	0.134	0.194
Adasyn Balanced vs. SMOTE Balanced	0.482	0.960	0.858
Adasyn Balanced vs. GAN Balanced	0.074	$$< $$ 0.001 *	$$< $$ 0.001 *
SMOTE Balanced vs. GAN Balanced	0.011	$$< $$ 0.001 *	$$< $$ 0.001 *

*Note* Pairwise comparisons between data generation methods were performed using Mann-Whitney U tests with a Bonferronicorrected significance level of 0.0083 (0.05 / 6 comparisons). *P*-values less than 0.0083 are considered statistically significant (*)

**Fig. 2:**
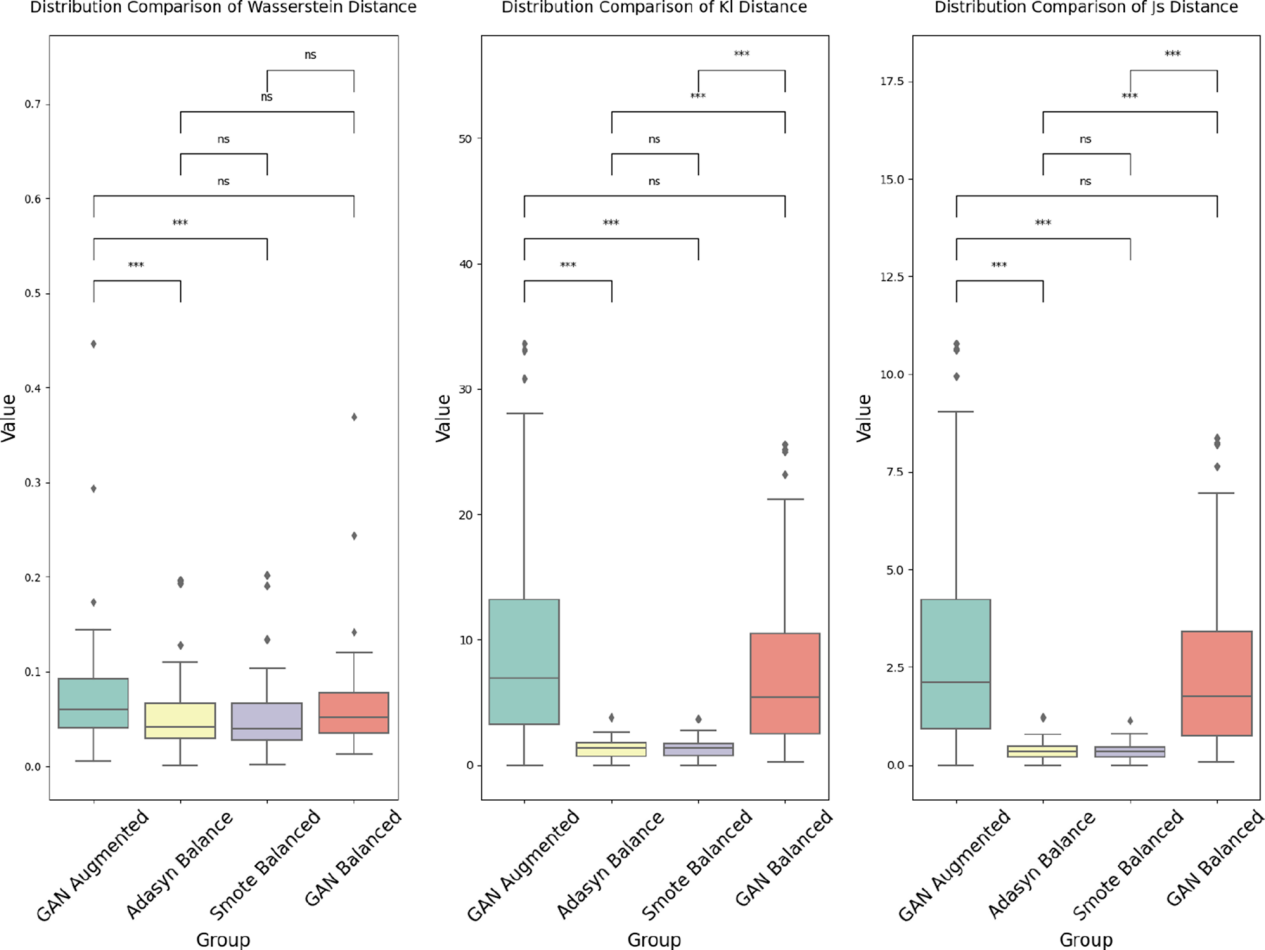
Comparison of data distribution similarity metrics across data generation methods. This figure presents box plots comparing the distributions of (**a**) Wasserstein distance, (**b**) kullback-leibler (KL) divergence, and (**c**) jensen-shannon (JS) divergence between the real data and the synthetic data generated by four different methods: GAN augmented, adasyn balanced, SMOTE balanced, and GAN balanced. The significance levels indicated as ***:${p}< 0.0083$, ns: not significant

In summary of the quantitative assessments, the Kruskal-Wallis tests show significant overall differences (${p}< 0.001$) across all three distance metrics for the four datasets. Pairwise comparisons (Table 6) indicate that the GAN Augmented dataset differs significantly (${p}<.0083$) from the Adasyn Balanced and SMOTE Balanced dataset in all metrics. The Adasyn Balanced and SMOTE Balanced datasets exhibit no significant differences from each other across all metrics. The GAN Balanced dataset differs significantly (${p}<.0083$) from both Adasyn Balanced and SMOTE Balanced in KL and JS Divergence, but not Wasserstein Distance (*p* = 0.074 and *p* = 0.011 respectively). Notably, the two GAN-based datasets (Augmented and Balanced) show no significant differences from each other in KL and JS Divergence (*p* > 0.05). The illustrated comparison of four datasets is shown in Fig. 2.

### Difference between datasets in prediction model performance

To rigorously evaluate the impact of different data generation methods on the predictive performance of IDH models, a one-way analysis of variance (ANOVA) was conducted to compare the model performance metrics across the five datasets: Original Training Data, GAN Augmented, Adasyn Balanced, SMOTE Balanced, and GAN Balanced dataset. Table 7 summarizes the results of the ANOVA tests, revealing statistically significant differences between the data generation methods across all performance metrics (${p}< 0.001$). Table 8 compares the mean and standard deviation of these performance metrics for each dataset, and Table 9 further details the pairwise comparisons.

**Table 7:** One-way ANOVA results for model performance metrics across different data generation methods: assessing statistical significance of inter-group differences

Performance Metric	F-value	*p*-value	Effect Size ($\omega^2$)
Accuracy	26.0 (4, 145)	0.001 *	0.400
F1-score	41.4 (4, 145)	0.001 *	0.519
ROC-AUC	44.5 (4, 145)	0.001 *	0.537
PR-AUC	33.4 (4, 145)	0.001 *	0.463

*Note* df = 4, 145 (degrees of freedom between and within groups); 𝜔^2^ = omega-squared effect size, representing the proportion of variance in the performance metric attributable to the data generation method. Effect sizes are interpreted as: small (𝜔^2^ ≥ 0.01), medium (𝜔^2^ ≥ 0.06), and large (𝜔^2^ ≥ 0.14). *P*-values are two-tailed. Significance levels (*: p < 0.05) are indicated

**Table 8:** Performance metrics comparison across different data generation methods: mean ($\pm$SD)

Method	Accuracy	F1-score	ROC-AUC	PR-AUC
Original Data	0.892$\pm$0.003	0.877$\pm$0.003	0.925$\pm$0.004	0.724$\pm$0.013
GAN Augmented	0.898$\pm$0.004	0.885$\pm$0.004	0.915$\pm$0.005	0.713$\pm$0.016
Adasyn Balanced	0.896$\pm$0.004	0.886$\pm$0.005	0.922$\pm$0.007	0.702$\pm$0.020
SMOTE Balanced	0.895$\pm$0.002	0.886$\pm$0.003	0.922$\pm$0.005	0.698$\pm$0.012
GAN Balanced	0.900$\pm$0.003	0.890$\pm$0.004	0.931$\pm$0.001	0.735$\pm$0.009

*Note* Performance under different data generation and balancing strategies is evaluated using Accuracy, F1-score, Area Under the ROC Curve (ROC-AUC), and Area Under the Precision-Recall Curve (PR-AUC). All values are presented as Mean ± Standard Deviation (SD), computed from 30 independent experimental runs for each method

**Table 9:** Pairwise comparison results of different data generation methods

Comparison groups	Mean difference	Cohen’s d	*p*-value	Significance
**A. Accuracy Comparisons**				
Original Data- GAN Augmented	0.006	1.878	0.001	*
Original Data- Adasyn Balanced	0.004	0.986	0.001	*
Original Data- SMOTE Balanced	0.003	1.216	0.001	*
Original Data- GAN Balanced	0.008	2.574	0.001	*
GAN Augmented - Adasyn Balanced	−0.003	−0.721	0.008	
GAN Augmented - SMOTE Balanced	−0.003	−1.015	0.001	*
GAN Augmented - GAN Balanced	0.002	0.528	0.049	
Adasyn Balanced - SMOTE Balanced	−0.000	−0.080	0.761	
Adasyn Balanced - GAN Balanced	0.005	1.250	0.001	*
SMOTE Balanced - GAN Balanced	0.005	1.764	0.001	*
**B. F1-score Comparisons**				
Original Data- GAN Augmented	0.008	2.147	0.001	*
Original Data- Adasyn Balanced	0.009	2.097	0.001	*
Original Data- SMOTE Balanced	0.009	2.866	0.001	*
Original Data- GAN Balanced	0.013	3.525	0.001	*
GAN Augmented - Adasyn Balanced	0.001	0.234	0.377	
GAN Augmented - SMOTE Balanced	0.001	0.377	0.156	
GAN Augmented - GAN Balanced	0.005	1.195	0.001	*
Adasyn Balanced - SMOTE Balanced	0.000	0.067	0.798	
Adasyn Balanced - GAN Balanced	0.004	0.800	0.003	*
SMOTE Balanced - GAN Balanced	0.003	0.954	0.001	*
**C. ROC-AUC Comparisons**				
Original Data- GAN Augmented	−0.010	−2.306	0.001	*
Original Data- Adasyn Balanced	−0.003	−0.506	0.059	
Original Data- SMOTE Balanced	−0.003	−0.607	0.024	
Original Data- GAN Balanced	0.006	2.323	0.001	*
GAN Augmented - Adasyn Balanced	0.007	1.279	0.001	*
GAN Augmented - SMOTE Balanced	0.007	1.404	0.001	*
GAN Augmented - GAN Balanced	0.016	4.304	0.001	*
Adasyn Balanced - SMOTE Balanced	−0.000	−0.018	0.945	
Adasyn Balanced - GAN Balanced	0.009	1.863	0.001	*
SMOTE Balanced - GAN Balanced	0.009	2.261	0.001	*
**D. PR-AUC Comparisons**				
Original Data- GAN Augmented	−0.011	−0.794	0.004	*
Original Data- Adasyn Balanced	−0.022	−1.331	0.001	*
Original Data- SMOTE Balanced	−0.026	−2.154	0.001	*
Original Data- GAN Balanced	0.011	1.010	0.001	*
GAN Augmented - Adasyn Balanced	−0.011	−0.598	0.027	
GAN Augmented - SMOTE Balanced	−0.015	−1.046	0.001	*
GAN Augmented - GAN Balanced	0.022	1.727	0.001	*
Adasyn Balanced - SMOTE Balanced	−0.004	−0.231	0.382	
Adasyn Balanced - GAN Balanced	0.033	2.141	0.001	*
SMOTE Balanced - GAN Balanced	0.037	3.571	0.001	*

*Note* Cohen’s d effect can be interpreted as: small (|𝑑| ≥ 0.2), medium (|𝑑| ≥ 0.5), and large (|𝑑| ≥ 0.8). Significance levels with Bonferroni correction: (* as p < 0.005)

The ANOVA results (Table 7) show significant F-values for Accuracy (F(4, 145) = 26.0, p $$< $$ 0.001,$$\omega^2$$ = 0.400), F1-score (F(4, 145) = 41.4, p$$< $$ 0.001,$$\omega^2$$ = 0.519), ROC-AUC (F(4, 145) = 44.5, *p*$$< $$ 0.001,$$\omega^2$$ = 0.537), and PR-AUC (F(4, 145) = 33.4, *p*$$< $$ 0.001,$$\omega^2$$ = 0.463).

Performance metrics comparison across datasets: Table 8 provides a detailed comparison of the average performance metrics for each data generation method. The updated pairwise comparisons (Table 9) reveal nuanced differences between the data generation techniques.

- Accuracy: The GAN Balanced dataset achieves the highest average accuracy (0.900), and all augmentation/balancing techniques significantly improve upon the Original Training Data (all *p*$$< $$0.001, Table 9A). For instance, GAN Balanced shows a mean difference of 0.008 compared to Original Data ($${p}< 0.001$$).
- F1-score: Similar to accuracy, GAN Balanced achieves the highest average F1-score (0.890). All augmentation/balancing methods significantly outperform the Original Training Data (all *p*$$< $$ 0.001, Table 9B). GAN Balanced shows the largest improvement over Original Data (Mean Difference = 0.013,$${p}< 0.001$$).
- ROC-AUC: The GAN Balanced dataset achieves the highest average ROC-AUC (0.931), significantly outperforming the Original Training Data (Mean Difference = 0.006, *p*$$< $$ 0.001) (Table 9C). Interestingly, the GAN Augmented dataset shows a significant *decrease* in ROC-AUC compared to the Original Training Data (Mean Difference =  $$-$$0.010, *p *$$< $$ 0.001). In contrast, the Adasyn Balanced and SMOTE Balanced datasets show no statistically significant difference from the Original Data at the corrected alpha level (*p* = 0.059 and *p* = 0.024, respectively).
- PR-AUC: GAN Balanced again shows the highest average performance (0.735) and a significant improvement over the Original Training data (Mean Difference = 0.011, *p*$\:<$ 0.001). However, unlike ROC-AUC, the GAN Augmented dataset shows a significant *decrease* compared to Original Data (Mean Difference =  $-$0.011, *p* = 0.004), while both Adasyn and SMOTE Balanced also show a significant decrease (Mean Differences = −0.022 and −0.026 respectively, both ${p}< 0.001$) (Table 9D).

**Fig. 3:**
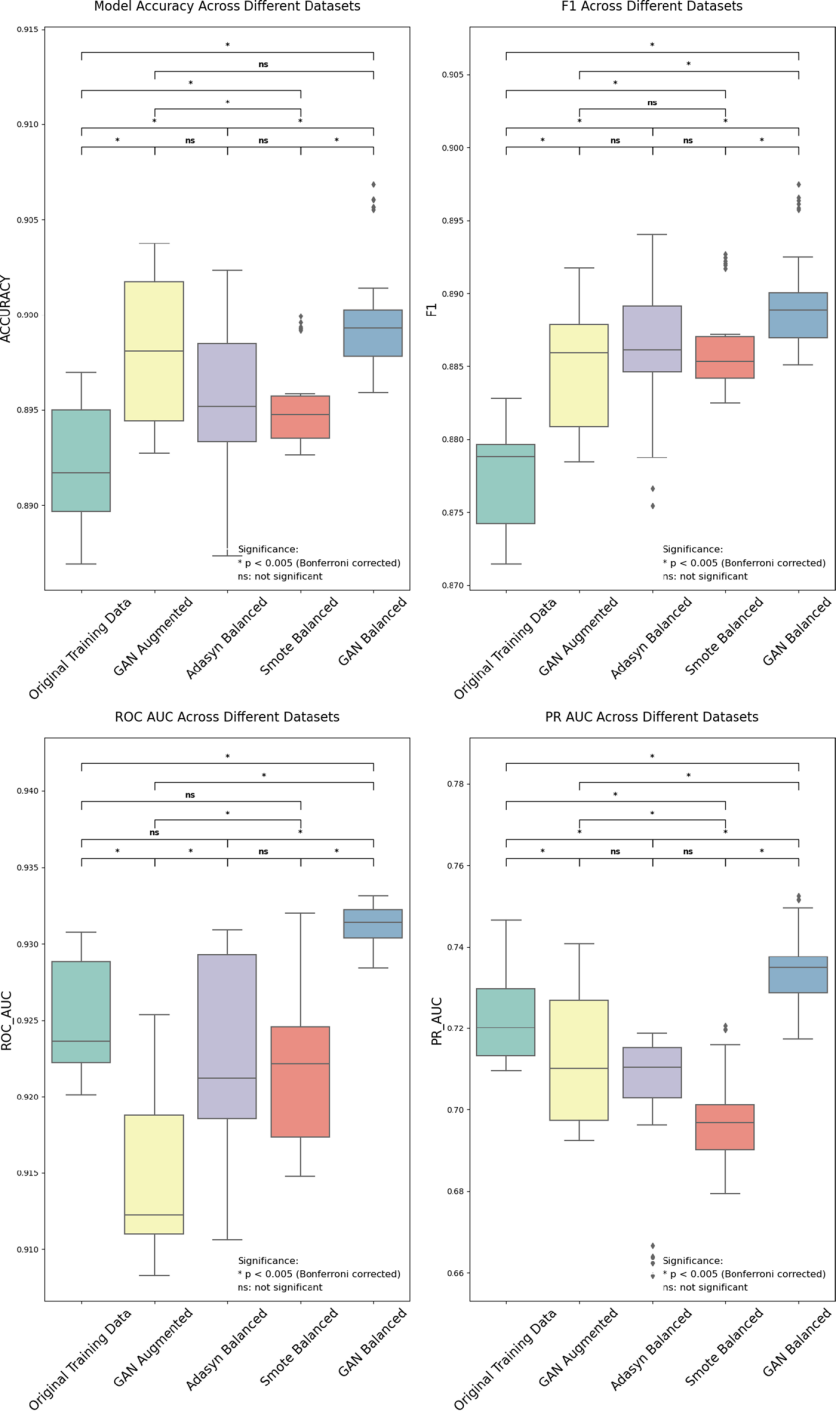
Boxplots comparing IDH prediction performance (accuracy, F1-score, ROC AUC, and PR AUC) of XGBoost models trained on the original dataset and datasets balanced using GAN augmentation, ADASYN, SMOTE, and the proposed GAN balanced method. Significance levels (*:*p*$$< $$ 0.005, ns: not significant) are indicated for pairwise comparisons

In summary, the GAN Balanced dataset consistently demonstrated the highest average performance across all four metrics. It showed statistically significant improvements compared to the Original Training Data (${p}< 0.001$ for all metrics). The GAN Augmented dataset yielded mixed results, improving Accuracy and F1-score but decreasing ROC-AUC and PR-AUC compared to the Original Data. The Adasyn Balanced and SMOTE Balanced datasets improved Accuracy and F1-score over the Original Data but resulted in significantly lower PR-AUC and showed no significant improvement in ROC-AUC. Pairwise comparisons among methods (Table 9) reveal numerous significant differences (${p}< 0.005$), particularly highlighting the superior performance of the GAN Balanced dataset, often with large effect sizes (e.g., Cohen’s d = 3.525 for F1-score vs Original Data). Figure 3 visually summarizes the performance distributions of the XGBoost models across the different data generation methods, complementing the statistical analyses presented in Tables 7,8, and 9.

### Features importance analysis

**Fig. 4:**
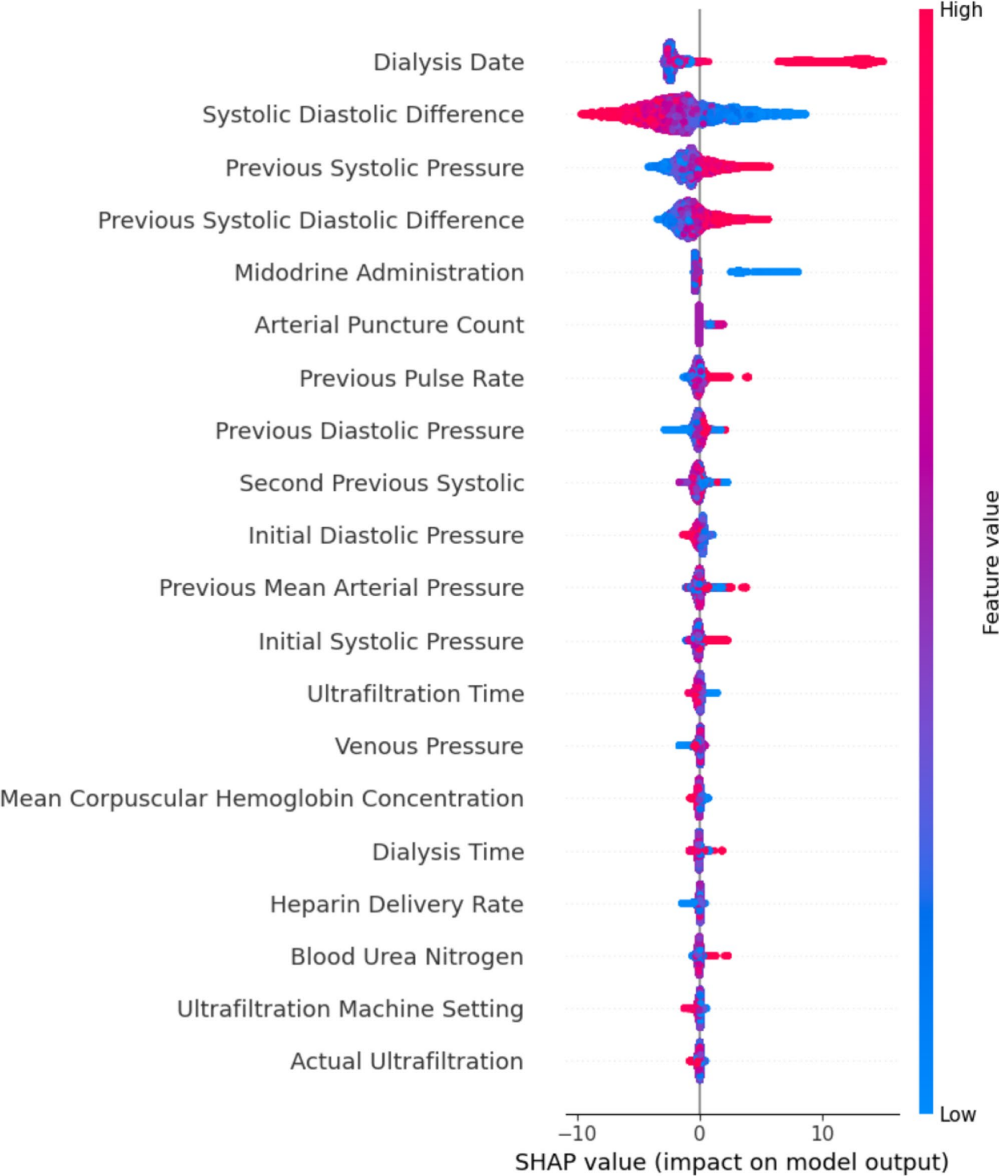
SHAP summary plot for IDH prediction model trained on GAN balanced dataset. The SHAP summary plot visualizes feature importances for the XGBoost model trained on the GAN balanced dataset. Features are ranked in descending order of importance based on mean absolute SHAP values. Each point represents a SHAP value for a feature and an instance, with color indicating feature value (red = high, blue = low) and horizontal position indicating SHAP value (impact on model output)

To elucidate the underlying factors driving IDH risk prediction and to gain clinically relevant insights, SHAP analysis was performed on the XGBoost model trained on the GAN Balanced dataset, which demonstrated the highest overall predictive performance (Fig. 4).

In summary, Dialysis Date has the highest mean absolute SHAP value, followed by Systolic Diastolic Difference and Previous Systolic Pressure. Other features include Previous Pulse Rate, Previous Diastolic Blood Pressure, Actual Ultrafiltration, Initial Diastolic Pressure, Second Previous Systolic Pressure, Dialysis Time, Ultrafiltration Volume, and baseline albumin-bcg, ranked in descending order of mean absolute SHAP values based on the analysis of the model trained with the GAN Balanced dataset. For Dialysis Date and systolic-diastolic difference, higher values (red points) are associated with positive SHAP values (increased predicted IDH risk). In contrast, for Previous Systolic Pressure and Previous Diastolic Blood Pressure, higher values (red points) are associated with negative SHAP values (decreased predicted IDH risk). Previous Pulse Rate shows higher values associated with positive SHAP values, while Previous Diastolic Blood Pressure shows lower values associated with positive SHAP values. Features like Actual Ultrafiltration, Dialysis Time, Ultrafiltration Volume, and baseline albumin-bcg have SHAP value distributions centered around zero, indicating less overall impact on the model’s output across the dataset.

## Discussion

This study provides compelling evidence for the effectiveness of a Generative Adversarial Network (GAN)-based data balancing strategy in significantly enhancing the performance of Intradialytic Hypotension (IDH) prediction models. By employing a conditional WGAN-GP, we aimed to address key challenges inherent in hemodialysis data, particularly the class imbalance often observed in single-center datasets and the need to capture complex, non-linear relationships within the data. Our analysis of a dataset encompassing 128,741 records from 40 chronic hemodialysis patients revealed typical risk profiles and a baseline IDH event rate of 14.85%, consistently reflected in the training (14.73%) and testing (15.21%) sets. While quantitative assessment of generated data showed trade-offs – traditional methods (ADASYN, SMOTE) appeared closer to the original distribution based on KL/JS divergence, whereas GAN methods exhibited greater distributional dissimilarity but potentially more diversity – the downstream predictive performance strongly favored the GAN-based approach. The GAN Balanced dataset consistently yielded the best results across all metrics, achieving statistically significant improvements over the original imbalanced data, notably in Accuracy (mean 0.900 vs 0.892,${p}< 0.001$) and PR-AUC (mean 0.735 vs 0.724,${p}< 0.001$), often with large effect sizes (e.g., Cohen’s d = 3.525 for F1-score). Interestingly, the GAN Augmented dataset showed mixed results, improving Accuracy/F1 but decreasing ROC-AUC, while ADASYN/SMOTE significantly reduced PR-AUC. SHAP analysis further identified clinically relevant predictors, with Dialysis Date emerging as most important (potentially capturing temporal effects), followed by hemodynamic indicators like Systolic Diastolic Difference and Previous Systolic Pressure. These findings collectively suggest that our GAN-based balancing framework not only improves predictive accuracy but also generates data that is helpful in deriving interpretable insights, paving the way for more reliable clinical decision support tools in hemodialysis care. The subsequent sections will delve deeper into the methodological considerations, the effectiveness of the balancing strategies, the interpretation of feature importance, and the broader clinical implications.

### Methodological considerations in data handling

Rigorous data handling was paramount in this study due to the time-dependent nature of clinical dialysis records, requiring careful consideration of data collection, splitting, preprocessing, and statistical analysis choices.

Incorporating multi-level data, including high-frequency signals from dialysis machines and potentially wearable devices alongside traditional clinical records and laboratory results, was a deliberate strategy employed in this study to enhance predictive capability for IDH. As highlighted in the literature, leveraging high-resolution, granular data sources can significantly improve the performance of predictive models in complex healthcare scenarios like hemodialysis [36]. Such data may capture subtle, transient physiological changes or machine dynamics preceding an IDH event that are often missed by lower-frequency data points (e.g., monthly labs or pre/post-dialysis measurements). Our approach aimed to harness this potential by integrating both static patient characteristics and dynamic intra-dialytic variables, seeking to create a richer, more informative feature set. While managing and effectively integrating these diverse data streams presents inherent challenges—including data fusion complexities, handling varying data frequencies, and sophisticated feature engineering—the potential benefits in capturing the complex temporal dynamics leading to IDH underscore the value of pursuing comprehensive data collection for developing more accurate and clinically relevant predictive models.

Following data collection, a strict date-based temporal split was employed, allocating the initial 75% of the chronological data period to training and the subsequent 25% to testing. This temporal partitioning is fundamental for preventing data leakage, where future information could inadvertently inform predictions about the past, ensuring a realistic evaluation of model performance over time. This setting resulted in an actual data record ratio closest to 75:25 among all feasible time cut points. While various train/test split ratios are used in medical machine learning [23, 24, 27], we retained a relatively large 25% test set primarily to enable a reliable comparison of the different data augmentation and balancing techniques (cWGAN-GP vs. SMOTE/ADASYN). A substantial test set provides more stable estimates of performance gains, minimizing the influence of random fluctuations. Furthermore, this temporal split strategy directly mirrors the intended clinical application—predicting future IDH risk for currently managed patients based on their historical data—rather than assessing generalization to entirely new patients, which would require a patient-level split.

Subsequent to splitting, feature scaling was performed. The choice of the Robust Tanh Scaler represents a specific methodological decision balancing robustness against potential information loss, which is particularly relevant given the nature of our clinical dataset and the requirements of the GAN model. Standard scaling methods, such as StandardScaler, can be heavily skewed by the outliers frequently encountered in physiological measurements or lab results, potentially distorting the input distribution for sensitive models like GANs. By using median and interquartile range for initial scaling, our approach aimed to mitigate this outlier influence effectively [50, 51]. The subsequent Tanh transformation ensured bounded inputs within (−1, 1), a common practice considered beneficial for stabilizing the training dynamics of generative adversarial networks [49]. We acknowledge, however, that this non-linear ‘squashing’ inherently compresses the range of features, especially values far from the median. This might lead to a loss of discriminative information compared to linear scaling methods, presenting a trade-off between outlier resilience, model stability, and information preservation. While this trade-off was deemed acceptable for the primary goal of robust GAN training in this study, the precise impact of this Scaler choice versus alternatives on downstream predictive performance warrants further comparative investigation in future work.

To further ensure the integrity of our evaluation and address potential data leakage concerns during preprocessing and augmentation, we strictly adhered to the sequence outlined in the Methods: splitting the data temporally before any other step, including scaling and augmentation. Specifically, the parameters for feature scaling (using the Robust Tanh Scaler) were fitted only on the training dataset and then applied consistently to transform both the training and test sets. Crucially, all data augmentation and balancing procedures, including the training of the GAN and the application of SMOTE/ADASYN, were performed exclusively on the already split and scaled training data. The final predictive models (XGBoost) were then trained on these processed training sets (original or augmented/balanced) and evaluated on the entirely separate and untouched test set, guaranteeing a fair assessment free from information contamination during the augmentation and modeling phases.

### Effectiveness of GAN-based balancing: distributional characteristics vs. predictive utility

The observation that GAN-based methods demonstrably enhance predictive performance compared to ADASYN and SMOTE, despite exhibiting lower distributional similarity to the original dataset according to multiple metrics, merits further examination. Indeed, quantitative analysis presented in Table 6 confirms that traditional interpolation-based techniques like ADASYN and SMOTE yield distributions with higher statistical similarity (lower divergence/distance) to the original dataset across Wasserstein, KL, and JS metrics. However, this closer resemblance in overall distribution statistics, or higher fidelity, did not translate into superior downstream predictive accuracy for the IDH prediction task. Conversely, GAN-based approaches consistently outperformed these traditional methods in critical predictive metrics such as accuracy and PR-AUC (Table 9 and Fig. 3), indicating greater utility despite the measured distributional differences. This apparent discrepancy underscores a crucial distinction: data quality measured by global distributional similarity metrics may differ significantly from data quality defined by its utility in enhancing the performance of a specific predictive model.

This apparent discrepancy highlights potential limitations of evaluation methods; both quantitative metrics (Wasserstein, KL, JS) and qualitative visualizations like t-SNE primarily assess overall structural similarity. They may not fully capture data characteristics crucial for downstream classification performance, such as the quality and distribution of samples near the decision boundary. Furthermore, the generation mechanisms differ fundamentally. Adasyn/SMOTE interpolate between existing minority samples, ensuring local similarity but potentially failing to capture complex manifold structures or generating ‘blurry’ samples in inappropriate regions [37]. Conversely, GANs aim to learn the underlying data-generating distribution [43]. Even if imperfectly matched to training dataset, a successful GAN might better capture intrinsic structures and complex feature correlations or generate samples that more effectively define the decision boundary. These high-utility samples, even if deviating somewhat in overall distribution, could provide greater value for training the classifier compared to simple interpolations [42].

Furthermore, the specific conditional Wasserstein GAN (cWGAN) architecture used in this study, enhanced with residual networks and spectral normalization, appears particularly adept at handling such imbalanced datasets. The generator’s deep residual structure likely mitigates the vanishing gradient problem, enabling the learning of complex mappings. At the same time, spectral normalization in the discriminator ensures Lipschitz continuity, stabilizing training and potentially preventing mode collapse [44]. This robust training process seems to produce new synthetic IDH instances that augment the minority class in a clinically relevant manner, potentially avoiding the over-generalization and noise amplification sometimes observed with SMOTE and ADASYN, especially in high dimensions [39–41]. Indeed, for imbalanced classification tasks like IDH prediction, generating synthetic samples that effectively improve the model’s discriminative power near the decision boundary might be more valuable than samples that perfectly mimic the overall distribution but offer less information for separating classes. Consequently, our cWGAN-generated samples appear to empower the XGBoost model to learn robust decision boundaries, improving generalization and the identification of critical IDH events.

Beyond performance, computational aspects were also considered. While GAN training involves an upfront investment, our specific configuration, including the deliberate use of a relatively large batch size (1024) primarily to enhance computational efficiency for method comparison, resulted in a relatively efficient training time (approximately 4 minutes, Sect.3.2) by leveraging GPU parallelism. Although large batch sizes can sometimes raise concerns about GAN training stability, we monitored the process closely; as illustrated by the loss curves in Supplementary, our training exhibited stable convergence after initial fluctuations, proceeding for approximately 1900 epochs without clear signs of divergence or mode collapse. This empirical observation suggests that, for our specific dataset and task, the batch size of 1024 represented a viable trade-off, balancing the practical need for efficient model evaluation with the achievement of stable GAN training. Importantly, once trained, generating synthetic samples with the GAN is computationally inexpensive (a single forward pass), contrasting with the potentially scaling kNN search cost in SMOTE/ADASYN for large datasets.

Therefore, the findings highlight the importance of evaluating synthetic data generation techniques based on their ultimate utility for the specific application, particularly in complex domains like medicine, as this may not always align perfectly with traditional measures of distributional fidelity.

### Rigorous dataset evaluation through multifactorial model comparison

This study employed a multifactorial experimental design, systematically varying XGBoost hyperparameters (n_estimators and max_depth) across a range of combinations while training and evaluating models on five distinct datasets (Original Training Data, GAN Augmented, Adasyn Balanced, SMOTE Balanced, and GAN Balanced dataset). This approach was specifically chosen over optimizing hyperparameters individually for each dataset version; our primary goal was not necessarily to achieve peak performance for any single configuration, but rather to assess the relative and robust effectiveness of the different data augmentation strategies (GAN vs. traditional methods) in improving predictive capabilities under consistent evaluation conditions, especially within the context of limited clinical data availability. Consequently, instead of per-dataset HPO, this structured approach was utilized. The same fixed set of hyperparameter configurations was consistently applied to train and evaluate XGBoost models based on each dataset version.

To rigorously assess the impact and robustness of these augmentation strategies across varying model complexities, ANOVA was employed to statistically compare model performance across the different dataset versions, considering the effects of the varying n_estimators and max_depth settings and their potential interactions. This structured evaluation across multiple configurations provides stronger evidence for the robustness of any observed performance differences between augmentation methods than relying on a single, potentially dataset-specific, optimized model could provide, aligning with recent trends in machine learning research for various applications [18, 20, 21, 65]. Indeed, the sensitivity of model performance to hyperparameter choices is well-documented [65], underscoring the importance of evaluating augmentation effects across a range of settings rather than attributing gains solely to potentially fortuitous hyperparameter optimization. By consistently applying the same range of parameter combinations to models trained on each dataset version, we aimed to demonstrate that the performance benefits associated with certain augmentation methods (e.g., GAN-based) are robust across different model complexities and represent an inherent value added by the data processing step itself, independent of fine-tuned hyperparameter choices. This approach is crucial for mitigating the risk that observed performance gains are merely artifacts of overfitting achieved through fine-tuning on a specific dataset version, complementing other measures taken such as the strict temporal train-test split and model regularization (e.g., reg_lambda = 1 and constraining max_depth$\leq$ 16).

As visually summarized in Fig. 3 and quantitatively detailed in our ANOVA results (Table 7), the dataset indeed had a significant impact on model performance, independent of the specific hyperparameters chosen, providing more substantial evidence for the effectiveness of GAN-based data balancing and its potential to improve the robustness of IDH prediction models. While alternative downstream models or more sophisticated automated HPO techniques might yield different peak performance levels, our focus on XGBoost with this structured parameter exploration provides a controlled and interpretable framework specifically designed to answer our core research question regarding the robust comparison of the data augmentation techniques themselves under consistent evaluation conditions.

While our analysis focused on two key XGBoost hyperparameters, future research could extend this approach by exploring a wider range of model parameters, investigating different model architectures (e.g., neural networks, support vector machines), or utilizing diverse datasets from additional healthcare institutions. Nevertheless, the robust performance observed for the GAN Balanced dataset across the tested spectrum of model configurations further strengthens the conclusion that the GAN-based approach provides a valuable and reliable method for enhancing IDH prediction, mitigating the risks associated with model selection bias, and improving the potential for real-world applicability.

### Interpretation of SHAP analysis and clinical implications

The SHAP analysis (Fig. 4) provides valuable insights into the factors driving IDH prediction. The prominence of Dialysis Date as the most crucial feature is notable, likely acting as a proxy for underlying temporal patterns rather than a direct causal factor. The ‘Dialysis Date’ feature, beyond the identified day-of-the-week effect [7], may also capture other more complex or long-term time-related information. This hypothesis was substantiated by our supplementary analysis (see Supplementary Material 1), where converting the ‘Dialysis Date’ to a simpler ‘Weekday’ feature resulted in a notable change in the feature importance hierarchy. This finding suggests that the original date feature encapsulates critical information beyond the weekly cycle—such as long-term trends that could reflect disease progression or seasonal variations—which is lost upon simplification. The inherent nature of such temporal data highlights the complexity of time features in clinical settings and the challenges associated with fully capturing all relevant temporal dynamics. This also constitutes an important observation of this study in terms of temporal feature engineering. Understanding ‘Dialysis Date’ as a composite of these factors is key to its interpretation.

Beyond the temporal features, the significant influence of hemodynamic indicators included Systolic Diastolic Difference, Previous Systolic Pressure, and Previous Diastolic Pressure aligns with clinical understanding. Larger pulse pressure (Systolic Diastolic Difference) increased predicted IDH risk, while higher preceding systolic and diastolic pressures decreased risk, reflecting their fundamental role in IDH pathophysiology. Higher Previous Pulse Rate is also associated with increased risk, potentially indicating compensatory responses. In contrast, several operational parameters (e.g., Dialysis Time, Ultrafiltration Volume) and some baseline or intermittent clinical measures showed relatively minor overall predictive effects in the SHAP analysis, suggesting dynamic physiological indicators and temporal patterns held greater immediate predictive power in our model context.

The directionality provided by SHAP values offers clinical guidance: features where higher values associate with positive SHAP values (e.g., Dialysis Date, Systolic Diastolic Difference, Previous Pulse Rate) point towards increased IDH risk according to the model, while those where higher values associate with negative SHAP values (e.g., Previous Systolic/Diastolic Pressure) suggest decreased risk. This feature importance hierarchy underscores the value of vigilant hemodynamic monitoring and considering temporal risk patterns (like day-of-week). These insights can inform risk stratification, guide personalized interventions, and contribute to the development of more effective clinical decision support systems (CDSS) for IDH management.

### Clinical implications and practical applications for IDH prediction

Accurate IDH prediction holds significant clinical importance due to the established association of IDH with severe adverse outcomes, including increased mortality, cardiovascular events, and reduced quality of life [3, 9–16]. Therefore, models offering enhanced predictive performance, as demonstrated by our GAN-balanced approach, present substantial potential clinical value for early detection and prevention.

Our IDH prediction model, enhanced by GAN-generated synthetic data, could be integrated into clinical workflows to facilitate targeted interventions. Potential applications include embedding within hemodialysis machines for real-time risk display based on dynamic data, or integration with EHR systems for comprehensive risk assessment using patient profiles, potentially underpinning CDSS recommendations (e.g., UF rate adjustments, preemptive measures) and automated alerts. Real-time alerts across various platforms could empower providers to act swiftly.

These applications enable proactive, data-driven interventions. Furthermore, the model’s utility could extend to patient-facing tools promoting education and adherence. Widespread adoption of such predictive frameworks holds promise for reducing IDH severity and associated complications, ultimately improving patient safety and quality of life in hemodialysis care [10]. This approach bridges predictive analytics with actionable clinical practice, offering a scalable path towards better patient outcomes.

## Conclusions

### Concluding remarks

This study demonstrates the significant potential of employing an enhanced conditional Wasserstein GAN framework for balancing complex, imbalanced hemodialysis datasets to improve Intradialytic Hypotension prediction. Our findings indicate that models trained on the GAN Balanced dataset achieved superior predictive performance, particularly in metrics sensitive to minority class detection like PR-AUC, compared to models trained on the original imbalanced data or data balanced using traditional SMOTE or ADASYN methods. While the GAN Augmented dataset showed mixed results, the overall success of the GAN Balanced approach highlights the effectiveness of GANs in generating high-utility synthetic data that captures relevant clinical patterns beyond simple distributional similarity. Furthermore, SHAP analysis provided valuable interpretability, identifying key hemodynamic indicators and temporal factors (represented by Dialysis Date) as critical predictors, offering actionable insights for clinical monitoring and risk stratification.

In summary, the main contributions of this study include:

- Validation of a cWGAN-GP based framework for effectively balancing complex, imbalanced hemodialysis data to predict IDH.
- Demonstration of superior predictive performance (especially PR-AUC) using the GAN Balanced dataset compared to baseline and traditional SMOTE/ADASYN methods.
- Highlighting that GANs can generate high-utility synthetic data for predictive tasks, even if standard distributional metrics differ from simpler methods.
- Identification of key, clinically relevant IDH predictors using SHAP analysis on the GAN-enhanced prediction model.

### Limitations and future directions

While this study highlights the strengths of GAN-based methodologies, certain limitations define the scope of our findings and suggest possibilities for future research. Primarily, the dataset originated from a single medical center, which, although providing rich data, limits the generalizability of the findings due to potential center-specific patient demographics and clinical practices. The complexity of the cWGAN model also warrants consideration regarding computational resources.

A more significant limitation, however, lies in the engineering of the Dialysis Date feature. In our primary model, this feature was treated as a monotonically increasing number. While it proved highly predictive, its predictive power likely stems from its role as a proxy for unmeasured, time-dependent confounding variables rather than a direct causal effect. Specifically, as suggested by clinical context, the monotonic increase of the date is likely correlated with the long-term deterioration of patient health—a common trajectory in chronic diseases that was not explicitly captured by other available clinical variables.

Crucially, this design choice poses a fundamental limitation on the model’s generalizability to future data. A model trained on a specific date range cannot be reliably applied to new time periods, as the feature values fall outside its learned distribution. This severely restricts the model’s utility for real-time clinical deployment. To investigate our framework’s robustness to this issue, a sensitivity analysis was performed in a supplementary experiment where Dialysis Date was replaced with a cyclical Weekday feature. The results (see Supplementary Material 1) demonstrated that our proposed GAN Balanced framework remained superior even without the influence of the monotonic date feature. This suggests that while the primary model’s temporal feature has deployment limitations, the core data balancing methodology itself is robust.

Furthermore, regarding model interpretability, while SHAP analysis was employed to enhance transparency, it is essential to acknowledge that relying on a single model (XGBoost) and a single explanation method (SHAP) for assessing feature importance has inherent limitations; importance rankings can vary with different models or methodologies, and thus our SHAP results are specific to the trained XGBoost model. Methodologically, our study did not include a systematic sensitivity analysis for key GAN hyperparameters (e.g., batch size, gradient penalty coefficient); while our chosen parameters yielded stable training and effective results, further exploration could identify optimal settings or robustness boundaries. Finally, the lack of external validation on independent datasets from different institutions means the model’s robustness across diverse settings remains to be confirmed.

A key future direction, aligning with our aim to develop adaptable frameworks, is to validate the proposed GAN-balancing methodology across multiple institutions. This involves assessing the framework’s effectiveness in enabling centers with potentially smaller datasets, but rich high-resolution data, to build accurate local IDH prediction models, thereby addressing limitations associated with applying large, heterogeneous datasets directly to independent sites. Such cross-center validation should test the core methodology’s adaptability, potentially accommodating variations in available feature sets while using the consistent GAN-based balancing and modeling approach [31–33].

Further research should also focus on extending predictions to longer-term patient outcomes, facilitating seamless clinical workflow integration via decision support tools, and exploring the synergistic potential of incorporating multi-modal data [20, 21].

To directly address the aforementioned limitation of the monotonic date feature, investigating more advanced methods for temporal feature engineering is a crucial avenue. This includes exploring more comprehensive combinations of cyclical features (e.g., month, season in conjunction with day-of-week) or integrating dedicated time series analysis methods to better capture multi-layered temporal effects.

Continued methodological investigations into alternative models, advanced balancing techniques, hybrid approaches [17–19, 45], and transfer learning, alongside deeper exploration of IDH pathophysiology informed by clinical expertise [15, 16], will be crucial for advancing the development of robust, generalizable, and clinically impactful IDH prediction tools.

## Electronic supplementary material

Below is the link to the electronic supplementary material.

Supplementary Material 1

## Data Availability

The datasets generated and/or analysed during the current study are not publicly available due to privacy and ethical restrictions imposed by the Institutional Review Board of Tainan Municipal An-Nan Hospital. However, data may be available from the corresponding author upon reasonable request and with permission of the IRB.

## Code availability

The code used for data analysis in this study is available from the corresponding author on reasonable request.

## Declarations

### Relevant guidelines and regulations

All methods were performed in accordance with the Declaration of Helsinki and relevant guidelines and regulations stipulated by the Institutional Review Board of Tainan Municipal An-Nan Hospital.

## Abbreviations

GAN: Generative adversarial networks
IDH: Intradialytic hypotension
ESRD: End stage renal disease
HD: Hemodialysis
SVM: Support vector machine
ROC: Receiver operating characteristic
AUC: Area under the curve
RNN: Recurrent neural network
SMOTE: Synthetic minority over-sampling technique
ADASYN: Adaptive synthetic sampling approach
cWGAN-GP: Conditional Wasserstein GAN with gradient penalty
IRB: Institutional review board
IQR: Interquartile range
ROC-AUC: Receiver operating characteristic curve area under curve
PR-AUC: Precision-recall area under the curve
WD: Wasserstein distance
KLD: Kullback-Leibler divergence
JSD: Jensen-Shannon divergence
ANOVA: Analysis of variance
GEE: Generalized estimating equations
EHR: Electronic health records
SHAP: SHapley additive exPlanations
BP: Blood pressure
UFR: Ultrafiltration rate
TP: True positive
TN: True negative
FP: False positive
FN: False negative
SD: Standard deviation
CV: Coefficient of variation
HPO: Hyperparameter optimization
kNN: k-Nearest neighbors
CDSS: Clinical decision support system
NA: Not available / not applicable
NS: Not significant
XGboost: eXtreme gradient boosting
LightGBM: Light gradient boosting machine
IRB: Institutional review board

## References

[1] Francis A, Harhay MN, Albert CM, Ong SLT, Ortiz A, Fogo AB, Fliser D, Roy-Chaudhury P, Fontana M, Nangaku M, Wanner C, Malik C, Hradsky A, Adu D, Bavanandan S, Cusumano A, Sola L, Ulasi I, Jha V. Chronic kidney disease and the global public health agenda: An international consensus. Nat Rev Nephrol. 2024;20(7):473–85. 10.1038/s41581-024-00820-6. 38570631 10.1038/s41581-024-00820-6

[2] Cheng H-T, Xiaoqi X, Lim PS, Hung K-Y. Worldwide epidemiology of diabetes-related end-stage renal disease, 2000–2015. Diabetes Care. 2020;44(1):89–97. 10.2337/dc20-1913. 33203706 10.2337/dc20-1913

[3] Hamrahian SM, Vilayet S, Herberth J, Fülüp T. Prevention of intradialytic hypotension in hemodialysis patients: Current challenges and future prospects. Int J Nephrol and Renovascular Disease. 2023;16. 10.2147/IJNRD.S245621. 10.2147/IJNRD.S245621 PMC10404053 37547077

[4] Kanbay M, Ertuglu LA, Afsar B, Ozdogan E, Siriopol D, Covic A, Basile C, Ortiz A. An update review of intradialytic hypotension: Concept, risk factors, clinical implications and management. Clin Kidney J. 2020;13(6):981–93. 10.1093/ckj/sfaa078. 33391741 10.1093/ckj/sfaa078 PMC7769545

[5] Nakagawa N. Seasonal variation and predictors of intradialytic hypotension. 44(11):1551–53. 10.1038/s41440-021-00730-1-34417558. 34417558

[6] Uchiyama K, Shibagaki K, Yanai A, Kusahana E, Nakayama T, Morimoto K, Washida N, Itoh H. Seasonal variation and predictors of intradialytic blood pressure decline: A retrospective cohort study. 44(11):1417–27. 10.1038/s41440-021-00714-1. 10.1038/s41440-021-00714-1 34331031

[7] Correa S, Guerra-Torres XE, Ravi KS, Mothi SS, Waikar SS, Causland FRM. Risk of intradialytic hypotension by Day of the Week in maintenance hemodialysis. ASAIO J (American Society For Artificial Internal Organs). 2022;68(6):865–73. 10.1097/MAT.0000000000001576. 10.1097/MAT.0000000000001576 PMC10157838 34494985

[8] Wu Z, Lan S, Chen C, Zhang X, Zhang Y, Chen S. Seasonal variation: A non-negligible factor associated with blood pressure in patients undergoing hemodialysis. 9. 10.3389/fcvm.2022.820483. 10.3389/fcvm.2022.820483 PMC8971928 35369290

[9] Kim SG, Yun D, Lee J, Kim YC, Kim DK, Oh KH, Joo KW, Kim YS, Han SS. Impact of intradialytic hypotension on mortality following the transition from continuous renal replacement therapy to intermittent hemodialysis. Acute And Crit Care. 2023;38(1):86–94. 10.4266/acc.2022.00948-36442470. PMC10030245 36442470

[10] Wang J, Yao J, Zhu X, Wang T, Jianda L, Wei Q, Xue J, Yuanhao W, You L. Impact of frequent intradialytic hypotension on quality of life in patients undergoing hemodialysis. BMC Nephrol. 2023;24(1):209. 10.1186/s12882-023-03263-637452301. 10.1186/s12882-023-03263-6 PMC10347841 37452301

[11] Liu Q, Wang W, Wu X, Lv J, Cai S, Li Y. Intra-dialytic blood pressure variability is a greater predictor of cardiovascular events in hemodialysis patients. BMC Nephrol. 2023;24(1):113. 10.1186/s12882-023-03162-w. 10.1186/s12882-023-03162-w PMC10134565 37101121

[12] Flythe JE, Xue H, Lynch KE, Curhan GC, Brunelli SM. Association of mortality risk with various definitions of intradialytic hypotension. J Am Soc Nephrol. 2015;26(3):724–34. 10.1681/ASN.2014020222. 10.1681/ASN.2014020222 PMC4341481 25270068

[13] Stefánsson BV, Brunelli SM, Cabrera C, Rosenbaum D, Anum E, Ramakrishnan K, Jensen DE, Stålhammar N-O. Intradialytic hypotension and risk of cardiovascular disease. Clin J Am Soc of Nephrol. 2014;9(12):2124–32. 10.2215/CJN.02680314. 10.2215/CJN.02680314 PMC4255399 25376764

[14] Shafi T, Sozio SM, Bandeen-Roche KJ, Ephraim PL, Luly JR, St. Peter WL, McDermott A, Scialla JJ, Crews DC, Tangri N, Miskulin DC, Michels WM, Jaar BG, Herzog CA, Zager PG, Meyer KB, Wu AW, Boulware LE. Predialysis systolic bp variability and outcomes in hemodialysis patients. J Am Soc Nephrol. 2014;25(4):799–809. 10.1681/ASN.2013060667. 10.1681/ASN.2013060667 PMC3968504 24385593

[15] Inrig JK, Oddone EZ, Hasselblad V, Gillespie B, Patel UD, Reddan D, Toto R, Himmelfarb J, Winchester JF, Stivelman J, Lindsay RM, Szczech LA. Association of intradialytic blood pressure changes with hospitalization and mortality rates in prevalent esrd patients. Kidney Int. 2007;71(5):454–61. https://doi.org/10/d4jrh6. 10.1038/sj.ki.5002077 PMC3149815 17213873

[16] Inrig JK, Patel UD, Toto RD, Szczech LA. Association of blood pressure increases during hemodialysis with 2-year mortality in incident hemodialysis patients: A secondary analysis of the dialysis morbidity and mortality wave 2 study. Am J Kidney Dis: The Off J Natl Kidney Foundation. 2009;54(5):881–90. https://doi.org/10/cjjnkz. 10.1053/j.ajkd.2009.05.012 PMC2767411 19643520

[17] Alzakari SA, Alhussan AA, Qenawy A-ST, Elshewey AM. Early detection of potato disease using an enhanced convolutional neural network-long short-term memory deep learning model. 10.1007/s11540-024-09760-x.

[18] Elshewey AM, Shams MY, Tawfeek SM, Alharbi AH, Ibrahim A, Abdelhamid AA, Eid MM, Khodadadi N, Abualigah L, Khafaga DS, Tarek Z. Optimizing hcv disease prediction in Egypt. The Hyoptgb Framew. 13(22):3439. 10.3390/diagnostics13223439. 10.3390/diagnostics13223439 PMC10670002 37998575

[19] Elshewey AM, Tawfeek SM, Alhussan AA, Radwan M, Abed AH. Optimized deep learning for potato blight detection using the waterwheel plant algorithm and sine cosine algorithm. 10.1007/s11540-024-09735-y.

[20] Alkhammash EH, Kamel AF, Al-Fattah SM, Elshewey AM. Optimized multivariate adaptive regression splines for predicting crude oil demand in Saudi Arabia. 2022(1). 8412895. 10.1155/2022/8412895.

[21] Alkhammash EH, Assiri SA, Nemenqani DM, Althaqafi RMM, Hadjouni M, Saeed F, Elshewey AM. Application of machine learning to predict covid-19 spread via an optimized bpso model. 8(6):457. 10.3390/biomimetics8060457. 10.3390/biomimetics8060457 PMC10604133 37887588

[22] Kumar A, Dhanka S, Singh J, Ali Khan A, Maini S. Hybrid machine learning techniques based on genetic algorithm for heart disease detection. 11:2450008. 10.1142/S2737599424500087.

[23] Dhanka S, Maini S. Random forest for heart disease detection: A classification approach. In: 2021 IEEE 2nd International Conference on Electrical Power and Energy Systems (ICEPES), p. 1–3. https://ieeexplore.ieee.org/document/9699506

[24] Dhanka S, Maini S. Multiple machine learning intelligent approaches for the heart disease diagnosis. IEEE EUROCON 2023 - 20th International Conference on Smart Technologies, p. 147–52. https://ieeexplore.ieee.org/document/10199080

[25] Dhanka S, Bhardwaj VK, Maini S. Comprehensive analysis of supervised algorithms for coronary artery heart disease detection. 40(7):13300. 10.1111//exsy.13300.

[26] Dhanka S, Maini S. Hyoptxgboost and hyoptrf: Hybridized intelligent systems using optuna optimization framework for heart disease prediction with clinical interpretations. 83(29):72889–937. 10.1007/s11042-024-18312-x.

[27] Sharma A, Dhanka S, Kumar A, Maini S. A comparative study of heterogeneous machine learning algorithms for arrhythmia classification using feature selection technique and multi-dimensional datasets. 6(3):035209. 10.1088/2631-8695/ad5d51.

[28] Maini S, Dhanka S. Hyper tuned rbf svm: A new approach for the prediction of the breast cancer. 2024 1st International Conference on Smart Energy Systems and Artificial Intelligence (SESAI), p. 1–4. https://ieeexplore.ieee.org/abstract/document/10599437

[29] Lin Y, Shi T, Kong G. Acute kidney injury prognosis prediction using machine learning methods: A systematic review. Kidney Med. 2025;7(1):100936. 39758155 10.1016/j.xkme.2024.100936 PMC11699606

[30] Kumar A, Sharma A, Dhanka S, Maini S. Machine learning for combating mental health stigma. In: Transforming neuropsychology and cognitive psychology with AI and Machine learning. IGI Global Scientific Publishing, p. 333–66. 10.4018/979-8-3693-9341-3.ch014.

[31] Gómez-Pulido JA, Gómez-Pulido JM, Rodríguez-Puyol D, Polo-Luque ML, Vargas-Lombardo M. Predicting the appearance of hypotension during hemodialysis sessions using machine learning classifiers. Int J Environ Res Pub Health And Public Health. 2021;18(5):2364. 10.3390/ijerph18052364. 10.3390/ijerph18052364 PMC7967733 33671029

[32] Zhang H, Wang L-C, Chaudhuri S, Pickering A, Usvyat L, Larkin J, Waguespack P, Kuang Z, Kooman JP, Maddux FW, Kotanko P. Real-time prediction of intradialytic hypotension using machine learning and cloud computing infrastructure. Nephrol Dialysis Transplant. 2023;38(7):1761–69. 10.1093/ndt/gfad070. 10.1093/ndt/gfad070 PMC10310501 37055366

[33] Lee H, Yun D, Yoo J, Yoo K, Kim YC, Kim DK, Oh KH, Joo KW, Kim YS, Kwak N, Han SS. Deep learning model for real-time prediction of intradialytic hypotension. Clin J Am Soc Nephrol. 2021;16(3):396–406. 10.2215/CJN.09280620 PMC8011016 33574056

[34] Duarte MP, Nóbrega OT, Vogt BP, Vieira FA, Mondini DR, Silva MZC, Disessa HS, Krug RR, Sant’Helena BRM, Bundchen DC, Bohlke M, Adamoli AN, Uchida MC, Avesani CM, Reboredo MM, Ribeiro HS. research in dialysis centers in brazil: Recruitment and implementation of the sarc-hd study. 47, 2024000910.1590/2175-8239-jbn-2024-0009en. 10.1590/2175-8239-JBN-2024-0009en PMC11755877 39776146

[35] Vandecasteele SJ, Kurella Tamura M. A patient-centered vision of care for esrd: Dialysis as a bridging treatment or as a final destination? JASN. 2014;25(8):1647–51. 10.1681/ASN.2013101082. 10.1681/ASN.2013101082 PMC4116069 24833125

[36] Ziegler J, Rush BNM, Gottlieb ER, Celi LA, Armengol de la Hoz MÁ. High resolution data modifies intensive care unit dialysis outcome predictions as compared with low resolution administrative data set. PLOS Digit Health. 2022;1(10):0000124. 10.1371/journal.pdig.0000124. 10.1371/journal.pdig.0000124 PMC9931257 36812632

[37] Zhao Y, Wong ZS-Y, Tsui KL. A framework of rebalancing imbalanced healthcare data for rare events’ classification: A case of look-alike sound-alike mix-up incident detection. J Healthc Eng. 2018;2018. 10.1155/2018/6275435-29951182. PMC5987310 29951182

[38] Haibo H, Bai Y, Garcia EA, Shutao L. Adasyn: Adaptive synthetic sampling approach for imbalanced learning. In: 2008 IEEE International Joint Conference on Neural Networks (IEEE World Congress on Computational Intelligence). 2008. p. 1322–28. 10.1109/IJCNN.2008.4633969. https://ieeexplore.ieee.org/document/4633969.

[39] Yuan X, Chen S, Zhou H, Sun C, Yuwen L. Chsmote: Convex hull-based synthetic minority oversampling technique for alleviating the class imbalance problem. Inf Sci (Ny). 2023;623. 10.1016/j.ins.2022.12.056.

[40] Soltanzadeh P, Hashemzadeh M. Rcsmote: Range-controlled synthetic minority oversampling technique for handling the class imbalance problem. Inf Sci. 2021;542:92–111. 10.1016/j.ins.2020.07.014.

[41] Hairani H, Widiyaningtyas T, Prasetya DD. Addressing class imbalance of health data: A systematic literature review on modified synthetic minority oversampling technique (smote) strategies. JOIV: Int J on Inf Visual. 2024;8(3):1310. 10.62527/joiv.8.3.2283.

[42] Ruben G, van Smeden Maarten TD, Van Calster B. The harm of class imbalance corrections for risk prediction models: Illustration and simulation using logistic regression. JAMIA. 2022;29(9):1525–34. 10.1093/jamia/ocac093 35686364. 10.1093/jamia/ocac093 PMC9382395 35686364

[43] Goodfellow IJ, Pouget-Abadie J, Mirza M, Xu B, Warde-Farley D, Ozair S, Courville A, Bengio Y. Generative adversarial NetworksGenerative adversarial networks. 2014. 10.48550/arXiv.1406.2661. http://arxiv.org/abs/1406.2661.

[44] Gulrajani I, Ahmed F, Arjovsky M, Dumoulin V, Courville A. Improved training of wasserstein gans. Improved Train of Wasserstein GANs. 2017. 10.48550/arXiv.1704.00028; http://arxiv.org/abs/1704.00028.

[45] Viji D, Dhanka S, Binda MB, Thomas M. Hybrid sto- iwgan method based energy optimization in fuel cell electric vehicles. Energy Convers Manag. 2024;305:118249. 10.1016/j.enconman.2024.118249.

[46] Brandt J, Lanzén E. A comparative review of SMOTE and ADASYN in imbalanced data classification. PhD thesis, Uppsala University, 2021. https://urn.kb.se/resolve?urn=urn:nbn:se:uu:diva-432162,.

[47] Sharma A, Dhanka S, Kumar A, Nain M, Dhanka B, Bhardwaj VK, Maini S, Arora AS. A systematic review on machine learning intelligent systems for heart disease diagnosis. 1–27. 10.1007/s11831-025-10271-2.

[48] Abdennour N, Ouni T, Amor NB. The importance of signal pre-processing for machine learning: The influence of data scaling in a driver identity classification. 2021 IEEE/ACS 18th International Conference on Computer Systems and Applications (AICCSA), pp. 1–6. https://ieeexplore.ieee.org/abstract/document/9686756

[49] Guimerà Cuevas F, Schmid H. Robust non-linear normalization of heterogeneous feature distributions with adaptive tanh-estimators. In: Proceedings of the 27th International Conference on Artificial Intelligence and Statistics, p. 406–14. PMLR. 10.56801/10.56801/v8.i.452. https://proceedings.mlr.press/v238/guimera-cuevas24a.html.

[50] He Y, Cui Z. Evaluating robust scale transformation methods with multiple outlying common items under irt true score equating. 44(4):296–310. 10.1177/0146621619886050. 10.1177/0146621619886050 PMC7262993 32536731

[51] Khoirunnisa A, Ramadhan NG. Improving malaria prediction with ensemble learning and robust scaler: An integrated approach for enhanced accuracy. 15(4):326–34. 10.20895/infotel.v15i4.1056.

[52] Arjovsky M, Chintala S, Bottou L. Wasserstein gan. arXiv preprint arXiv:1701.07875. 2017.

[53] Chawla NV, Bowyer KW, Hall LO, Kegelmeyer WP. Smote: Synthetic minority over-sampling technique. 16:321–57. 10.1613/jair.953.

[54] Dhanka S, Maini S. A hybridization of xgboost machine learning model by optuna hyperparameter tuning suite for cardiovascular disease classification with significant effect of outliers and heterogeneous training datasets. 420:132757. 10.1016/j.ijcard.2024.132757. 10.1016/j.ijcard.2024.132757 39615697

[55] Salimans T, Goodfellow I, Zaremba W, Cheung V, Radford A, Chen X. Improved techniques for training gans. Adv Neural Inf Process Syst. 2016.

[56] Borji A. Pros and cons of gan evaluation measures. Comput Vision Image Underst. 2019;179:41–65.

[57] Kullback S, Leibler RA. On information and sufficiency. Ann Math Stat. 1951;22(1):79–86.

[58] Joyce JM. Kullback-leibler divergence. Kullback-Leibler Divergence. 2011. 10.1007/978-94-007-0309-6.

[59] Endres DM, Schindelin JE. A new metric for probability distributions. IEEE Trans On Inf Theory. 2003;49(7):1858–60.

[60] Lin J. Divergence measures based on the shannon entropy. IEEE Trans On Inf Theory. 1991;37(1):145–51.

[61] Kruskal WH, Wallis WA. Use of ranks in one-criterion variance analysis. J Am Stat Assoc. 1952;47(260):583–621.

[62] Mann HB, Whitney DR. On a test of whether one of two random variables is stochastically larger than the other. Ann Math Stat. 1947;50–60.

[63] Bonferroni CE. Teoria statistica delle classi e calcolo delle probabilita. Pubblicazioni del R Istituto Superiore di Scienze Economiche e Commericiali di Firenze. 1936;8:3–62.

[64] Chen X, Duan Y, Houthooft R, Schulman J, Sutskever I, Abbeel P. Infogan: Interpretable representation learning by information maximizing generative adversarial nets. InfoGAN: Interpretable Represent Learn By Inf Maximizing Generative Adversarial Nets. 2016. 10.48550/arXiv.1606.03657. http://arxiv.org/abs/1606.03657.

[65] Srinivas P, Katarya R. Hyoptxg: Optuna hyper-parameter optimization framework for predicting cardiovascular disease using xgboost. Biomed Signal Process Control. 2022;73:103456. 10.1016/j.bspc.2021.103456.

[66] Cohen J. Statistical Power analysis for the behavioral sciences. 2nd. Routledge. 10.4324/9780203771587.

[67] Sawilowsky SS. New effect size rules of thumb. 8:597–99. 10.56801/10.56801/v8.i.452.

[68] Lundberg SM, Lee S-I. A unified approach to interpreting model predictions. Proceedings of the 31st International Conference on Neural Information Processing Systems. NIPS’17. Red Hook, NY, USA: Curran Associates Inc; 2017. p. 4768–77. 10.48550/arXiv.1705.0787.

