# Supplementary material for: A holistic framework for intradialytic hypotension prediction using generative adversarial networks-based data balancing
Source: BMC Med Inform Decis Mak. 2025 Jul 10;25:257. doi: 10.1186/s12911-025-03094-5 (PMC12243283; doi:10.1186/s12911-025-03094-5)
Supplement: Supplementary file 1 — Supplementary Material 1 [file 12911_2025_3094_MOESM1_ESM.pdf]

Supplementary Material

1 Training Loss Curse

The training process loss curve were shown in Figure 1. Based on the loss curves, the training process can be divided into three distinct phases. In Phase 1 (0-500 iterations), the generator loss (orange line) fluctuates with peaks reaching approximately 4.5, while the discriminator loss (blue line) varies between -4.0 and 1.0. Between iterations 500 and 1000 (Phase 2: Transition Phase), the loss curves for both the generator and discriminator show reduced oscillations compared to Phase 1, with amplitudes decreasing. After 1000 iterations (Phase 3: Stable Convergence), the discriminator loss stabilizes around 1.2, and the generator loss converges to approximately -1.8, with minimal fluctuations observed in both curves. An early stopping strategy was applied, halting training after approximately 1800 iterations when the discriminator loss plateaued. The average training time for the computational infrastructure used was approximately 4 minutes.

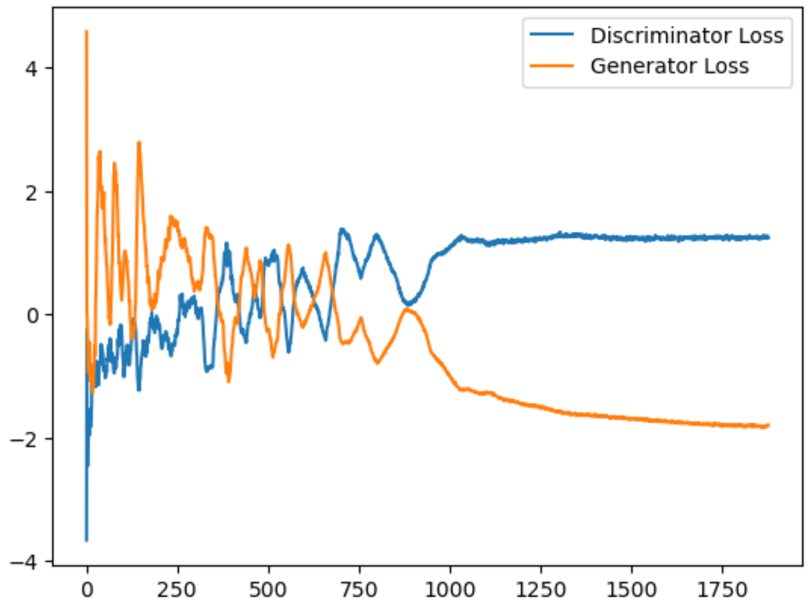

Figure 1: The blue line represents the discriminator loss, while the orange line represents the generator loss. The horizontal axis denotes the number of training iterations, and the vertical axis indicates the loss value. The training parameter settings are as follows: batch size = 1024, generator learning rate = 0.001, discriminator learning rate = 0.002, and a gradient penalty coefficient (λ_{GP}) of 5 was used to enforce the Lipschitz constraint as per the WGAN-GP framework. The total number of training epochs is 20, and the figure illustrates the training process over approximately 1800 iterations.

2 Variable Definitions

The Table 1 illustrate the detail of clincial definition of variables included in this research.

Table 1: Variable Definitions

Name	Definition
**Patient Characteristics Data**	
Male	Gender of the patient
Age	Age of the patient
Diabetes Mellitus Status	History of diabetes mellitus
Hypertension Status	History of hypertension
Cardiothoracic Ratio Binary	Cardiothoracic ratio over 0.5 or not
Midodrine Administration	Whether Midodrine was administered
**Nursing Records Data**	
Venous Needle Gauge	Gauge of the venous puncture needle
Arterial Needle Gauge	Gauge of the arterial puncture needle
Fall Risk Score	Fall Risk Assessment Score
Erythropoietin Dosage	Dosage of erythropoietin for anemia management
Dialysate Calcium Concentration	Concentration of calcium in the dialysate
Oral Fluid Intake	Indicator whether the patient ingested food during dialysis
Dry Weight	Ideal post-dialysis (dry) weight
Pre Dialysis Weight Net	Net body weight before dialysis
Ultrafiltration Machine Setting	Preset ultrafiltration volume on the machine
Post Dialysis Weight Net	Net body weight after dialysis
Actual Ultrafiltration	Actual volume of ultrafiltration achieved
**Hemodialysis Machine Data**	
Venous Pressure	Loop venous pressure (mmHg)
Arterial Pressure	Loop arterial pressure (mmHg)
Temperature	Temperature as recorded by the machine (°C)
Actual Blood Flow	Set blood flow rate, per dialysis prescription
Treatment Temperature	Dialysis prescriptionset treatment temperature (°C)
Actual Temperature	Actual blood temperature (°C)
Dialysate Conductivity	Conductivity of dialysate
Ultrafiltration Rate	Fluid removal rate (liter/hr)
Ultrafiltration Target	Target ultrafiltration volume (liter/hr); typically, a weight change within 35% of body weight is considered appropriate
Ultrafiltration Volume	Accumulated ultrafiltration volume (liter) at the current time
Ultrafiltration Time	Ultrafiltration Elapsed Time (minutes)

(Continued on next page)

Name	Definition
Dialysate Flow Rate	Dialysate flow rate (ml/min)
Cumulative Dialyate volume	Cumulative Dialysate volume(ml)
Target Sodium Concentration	Prescribed target sodium concentration (mEq/L); may be reduced in intradialytic hypertension
Bicarbonate Concentration	Prescribed bicarbonate concentration (mg/dl)
Effective Blood Flow	Effective blood flow rate (ml/min)
Ultrafiltration Profile	Mode of ultrafiltration
Sodium Profile	Sodium profile in the dialysis process
Heparin Delivery Rate	Anticoagulant (heparin) dose rate (ml/hr)
Arterial Puncture Count	Arterial puncture count
Venous Puncture Count	Venous Puncture Count
**Laboratory Data**	
Alkaline Phosphatase	Serum alkaline phosphatase level
Alanine Aminotransferase	Serum alanine aminotransferase level
Albumin Bcg	Serum albumin level
Blood Urea Nitrogen	Blood Urea Nitrogen level
Carbon Dioxide	Carbon dioxide level from blood draw
Creatinine	Serum creatinine level
Mean Corpuscular Volume	Mean corpuscular volume
Glomerular Filtration Rate	Estimated glomerular filtration rate
Glucose AC	Fasting blood glucose level
Hemoglobin	Hemoglobin level
Hematocrit	Hematocrit level
Potassium	Serum potassium level
Calcium	Serum calcium level
Sodium	Serum sodium level
Phosphorus	Serum phosphorus level
Platelet Count	Platelet count
Red Blood Cell Count	Red blood cell count
White Blood Cell Count	White blood cell count
Mean Corpuscular Hemoglobin (MCH)	Mean corpuscular hemoglobin value from blood test (MCH)
Mean Corpuscular Hemoglobin Concentration (MCHC)	Mean corpuscular hemoglobin concentration value from blood test (MCHC)
Mean Corpuscular Volume (MCV)	Mean corpuscular volume (MCV) value from blood test
Sodium	Serum sodium level
Phosphorus	Serum phosphorus level
Platelet Count	Platelet count
Red Blood Cell Count (RBC)	Red blood cell count (RBC) value from blood test
Red Cell Distribution Width (RDW)	Red blood cell distribution width (RDW) value from blood test

(Continued on next page)

Name	Definition
White Blood Cell Count (WBC)	White blood cell count (WBC) value from blood test
Initial Systolic Pressure	Systolic blood pressure at the start of the dialysis session
Initial Diastolic Pressure	Diastolic blood pressure at the start of the dialysis session
Systolic Diastolic Difference	Difference between systolic and diastolic pressure during dialysis
Previous Systolic Pressure	Systolic blood pressure from the previous measurement point
Previous Mean Arterial Pressure	Mean arterial pressure from the previous measurement point
Previous Diastolic Pressure	Diastolic blood pressure from the previous measurement point
Previous Pulse Rate	Pulse rate from the previous measurement point
Previous Systolic Diastolic Difference	Difference between previous systolic and diastolic pressure
Second Previous Systolic	Systolic blood pressure from two measurement points prior
Ocare Heart Rate Mean	Mean heart rate measured by wearable device between two measurement points
Ocare Heart Rate Cv	Coefficient of variation of heart rate measured by wearable device between two measurement points
Ocare Oxygen Saturation Mean	Mean SpO2 measured by wearable device between two measurement points
Ocare Oxygen Saturation Cv	Coefficient of variation of SpO2 measured by wearable device between two measurement points

Source: Hemodialysis Machine Data, Laboratory Data, Nursing Records, Patient Characteristics Data

3 Supplementory Experiment

Information about an additional experiment, beyond the main experiment, is presented in this supplementary material. This supplementary experiment uses the same dataset as the main experiment. The original variable Dialysis Date is converted into the Weekday, then data is segmented according to the original experimental steps (using the same data segmentation method as the Dialysis Date), and standardized. Using the Train dataset, GAN model training is performed using the same algorithm as the original experimental framework, and GAN augmented, ADASYN balanced, SMOTE balanced, and GAN balanced Datasets are created. Subsequently, multiple predictions are made using the same parameter combinations as the original experiment, and the results are statistically analyzed. For detailed research details, please refer to the experimental framework and related settings in the Section 'Material and Method' .

To evaluate the impact of different data generation methods on model performance, a one-way analysis of variance (ANOVA) was conducted. The results, as summarized

in Table 2, indicated that the choice of data generation method yielded statistically significant differences across all key performance metrics. Specifically, a significant effect was observed for Accuracy (p < .001), F1-Score (p < .001), ROC-AUC (p < .001), and PR-AUC (p < .001).

The detailed performance metrics for each method are presented in Table 3. Among the strategies, the GAN Balanced approach yielded the most robust performance. This method not only achieved high scores in Accuracy (0.903 ± 0.005) and F1-Score (0.891 ± 0.005) but also obtained the highest values for ROC-AUC (0.937 ± 0.004) and PR-AUC (0.751 ± 0.014). Comparatively, other data balancing techniques such as Smote and Adasyn also showed improved performance over the baseline model. Conversely, the GAN Augmented strategy did not yield a performance improvement, with its metrics generally falling below the baseline. The box plot was shown in Figure 2.

The outcomes of this supplementary experiment were highly consistent with the primary results. The analysis confirmed that the GAN Balanced method again achieved the best and most well-rounded performance. Although its F1-Score showed no statistical difference compared to traditional balancing methods, it maintained its statistically significant superiority in both ROC-AUC and PR-AUC over all other tested approaches.

Taken together, the findings from both the primary and supplementary experiments suggest that data balancing techniques are highly effective for model optimization, with the GAN Balanced method consistently emerging as the most effective strategy.

Table 2: One-Way ANOVA Results for Model Performance Metrics Across Different Data Generation Methods: Assessing Statistical Significance of Inter-group Differences

**Performance Metric**	**F-value**	**p-value**	**Effect Size (ω^2)**
Accuracy	21.1 (4, 145)	< 0.001 *	0.348
F1-Score	30.7 (4, 145)	< 0.001 *	0.442
ROC-AUC	43.2 (4, 145)	< 0.001 *	0.529
PR-AUC	40.5 (4, 145)	< 0.001 *	0.513

Note: df = 4, 145 (degrees of freedom between and within groups); ω^2 = omega-squared effect size, representing the proportion of variance in the performance metric attributable to the data generation method. Effect sizes are interpreted as: small (ω^2 ≥ 0.01), medium (ω^2 ≥ 0.06), and large (ω^2 ≥ 0.14). P-values are two-tailed. Significance levels (*: p < 0.05) are indicated.

Furthermore, to interpret the behavior of the model from this supplementary experiment, a SHAP (SHapley Additive exPlanations) analysis was performed to evaluate feature importance and impact, as shown in Figure 3. The importances changed after transformation of 'Dialysate Date' to 'Weekday'. This indicated that some hidden features captured by the dialysis date include the day of the week, disease deterioration, and season could be loss after transformation.

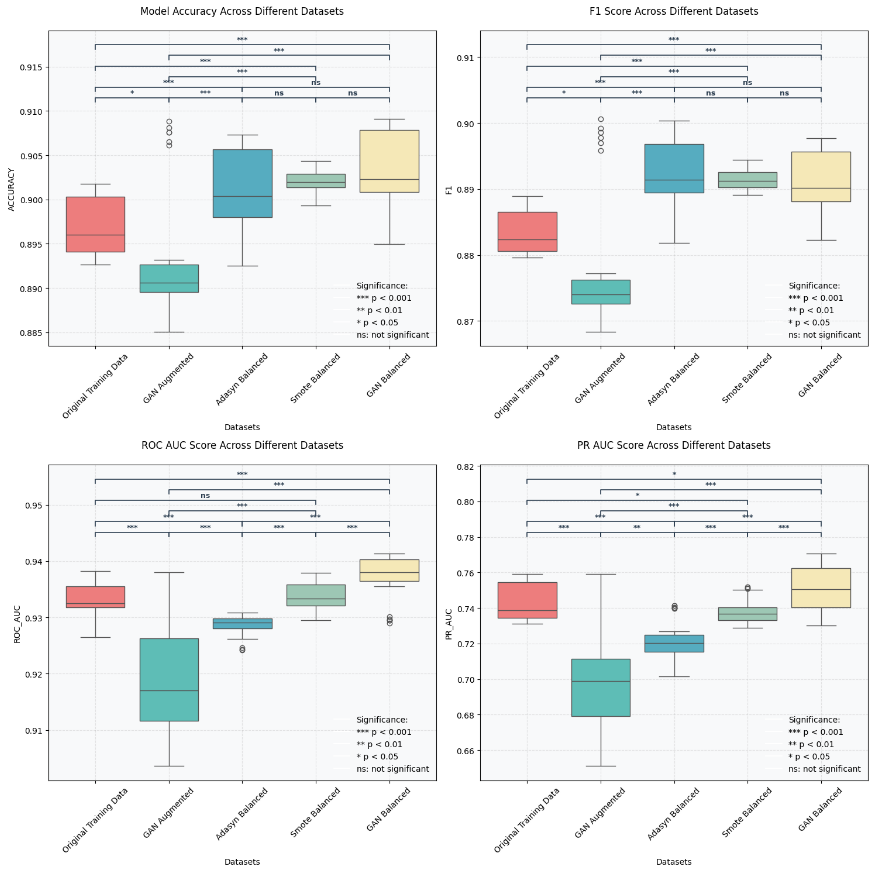

Figure 2: Boxplots comparing IDH prediction performance (Accuracy, F1-Score, ROC AUC, and PR AUC) of XGBoost models trained on the Original dataset and datasets balanced using GAN Augmentation, ADASYN, SMOTE, and the proposed GAN Balanced method. Significance levels (* : p < 0.005, ns: not significant) are indicated for pairwise comparisons.

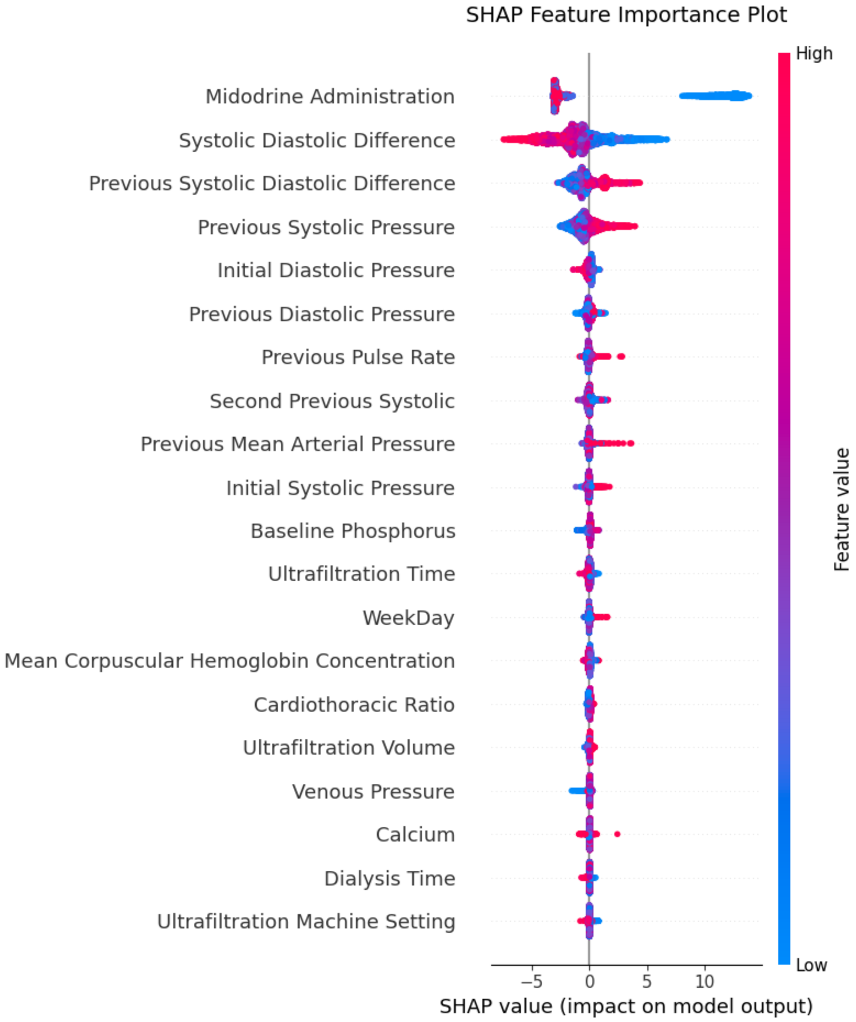

Figure 3: SHAP Summary Plot for IDH Prediction Model Trained on GAN Balanced Dataset. The SHAP summary plot visualizes feature importances for the XGBoost model trained on the GAN Balanced dataset. Features are ranked in descending order of importance based on mean absolute SHAP values. Each point represents a SHAP value for a feature and an instance, with color indicating feature value (red = high, blue = low) and horizontal position indicating SHAP value (impact on model output).

Table 3: Performance Metrics Comparison Across Different Data Generation Methods: Mean (±SD)

Method	Accuracy	F1-Score	ROC-AUC	PR-AUC
Original Training Data	0.897±0.003	0.884±0.003	0.933±0.004	0.744±0.010
GAN Augmented	0.893±0.007	0.878±0.010	0.919±0.011	0.700±0.033
Adasyn Balanced	0.901±0.005	0.892±0.006	0.929±0.002	0.721±0.012
Smote Balanced	0.902±0.001	0.891±0.002	0.934±0.002	0.738±0.007
GAN Balanced	0.903±0.005	0.891±0.005	0.937±0.004	0.751±0.014

Note: Performance under different data generation and balancing strategies is evaluated using Accuracy, F1-Score, Area Under the ROC Curve (ROC-AUC), and Area Under the Precision-Recall Curve (PR-AUC). All values are presented as Mean ± Standard Deviation (SD), computed from 30 independent experimental runs for each method.
